# Modulation of Wnt/BMP pathways during corneal differentiation of hPSC maintains ABCG2-positive LSC population that demonstrates increased regenerative potential

**DOI:** 10.1186/s13287-019-1354-2

**Published:** 2019-08-05

**Authors:** Meri Vattulainen, Tanja Ilmarinen, Laura Koivusalo, Keijo Viiri, Heidi Hongisto, Heli Skottman

**Affiliations:** 1 Faculty of Medicine and Health Technology, Tampere University, Arvo Ylpön katu 34, 33520 Tampere, Finland; 2 Tampere Center for Child Health Research, Faculty of Medicine and Health Technology, Tampere University, Tampere, Finland; 3 Department of Ophthalmology, Institute of Clinical Medicine, University of Eastern Finland, Kuopio, Finland

**Keywords:** Human pluripotent stem cells, Stem cell differentiation, Limbal stem cells, Stem cell hierarchy, Stem cell maintenance, Wnt signaling, ABCG2, Limbal stem cell deficiency

## Abstract

**Background:**

The differentiation of corneal limbal stem cells (LSCs) from human pluripotent stem cells (hPSCs) has great power as a novel treatment for ocular surface reconstruction and for modeling corneal epithelial renewal. However, the lack of profound understanding of the true LSC population identity and the regulation of LSC homeostasis is hindering the full therapeutic potential of hPSC-derived LSCs as well as primary LSCs.

**Methods:**

The differentiation trajectory of two distinct hPSC lines towards LSCs was characterized extensively using immunofluorescence labeling against pluripotency, putative LSC, and mature corneal epithelium markers. Cell counting, flow cytometry, and qRT-PCR were used to quantify the differences between distinct populations observed at day 11 and day 24 time points. Initial differentiation conditions were thereafter modified to support the maintenance and expansion of the earlier population expressing ABCG2. Immunofluorescence, qRT-PCR, population doubling analyses, and transplantation into an ex vivo porcine cornea model were used to analyze the phenotype and functionality of the cell populations cultured in different conditions.

**Results:**

The detailed characterization of the hPSC differentiation towards LSCs revealed only transient expression of a cell population marked by the universal stemness marker and proposed LSC marker ABCG2. Within the ABCG2-positive population, we further identified two distinct subpopulations of quiescent ∆Np63α-negative and proliferative ∆Np63α-positive cells, the latter of which also expressed the acknowledged intestinal stem cell marker and suggested LSC marker LGR5. These populations that appeared early during the differentiation process had stem cell phenotypes distinct from the later arising ABCG2-negative, ∆Np63α-positive third cell population. Importantly, novel culture conditions modulating the Wnt and BMP signaling pathways allowed efficient maintenance and expansion of the ABCG2-positive populations. In comparison to ∆Np63α-positive hPSC-LSCs cultured in the initial culture conditions, ABCG2-positive hPSC-LSCs in the novel maintenance condition contained quiescent stem cells marked by p27, demonstrated notably higher population doubling capabilities and clonal growth in an in vitro colony-forming assay, and increased regenerative potential in the ex vivo transplantation model.

**Conclusions:**

The distinct cell populations identified during the hPSC-LSC differentiation and ABCG2-positive LSC maintenance may represent functionally different limbal stem/progenitor cells with implications for regenerative efficacy.

**Electronic supplementary material:**

The online version of this article (10.1186/s13287-019-1354-2) contains supplementary material, which is available to authorized users.

## Background

The constant homeostatic regeneration of the human corneal epithelium (CE) is maintained by limbal stem cells (LSCs) that reside in their specific niche structures in the palisades of Vogt of the limbus [1]. Disturbances in the renewal process due to LSC dysfunction or loss manifest as a clinical condition called limbal stem cell deficiency (LSCD) that may in unilateral cases be treated with autologous cultured limbal epithelial transplantation (CLET) [2]. On the other hand, differentiation of LSCs from human pluripotent stem cells (hPSCs), including both human embryonic stem cells (hESCs) and induced pluripotent stem cells (hiPSCs), represents a promising therapy option for patients suffering from the bilateral condition [3, 4].

One of the most critical current challenges in the field of LSC therapies, with both primary and hPSC-derived cells, is the identification and maintenance of the clinically relevant LSC population. No specific single marker has been discovered to fill this gap. Thus, the phenotypic identification of LSCs currently relies on the expression of several stemness-related markers, combined with the absence of cytokeratins (CKs) 3 and 12 that mark terminally differentiated CE [5]. The tumor protein p63 is currently acknowledged as the classical identifier of colony-forming LSCs with proven clinical relevance and thus serves as a hallmark of high-quality CLET transplants [6, 7]. Moreover, among different p63 isoforms, ∆Np63α has been found to be the most abundantly expressed by LSCs [8, 9]. Other suggested LSC markers include ABCB5, BMI1, Frizzled-7, C/EBPδ, and CK15 [10–13]. In addition, studies in mice have pointed to ABCG2 being a universal marker of stemness in several tissues [14] and describing a slow-cycling subpopulation of colony-forming primary LSCs in humans [15, 16]. Thus, ABCG2 has been widely acknowledged as a marker for putative LSCs in vivo [5]*.*

Especially in the development of hPSC-based therapies, the potential presence of undifferentiated (UD) cells with tumorigenic potential among the transplantable cells raises an obvious safety concern. Markers that allow purification of the clinically relevant cell material by sorting would significantly promote both the safety and efficacy of the future treatments [17]. However, this issue is complicated by the fact that a low level of pluripotency markers is also expressed in the primary human limbal epithelium [18], and some of these markers are critically involved in the regulation of stemness [19]. A thorough understanding of the LSC differentiation hierarchy and the functional roles of various LSC-related markers is required to be able to address these questions. Recently, Bojic et al. identified two novel corneal cell surface proteins in primary cultured LSCs, namely, CD200, which marks a small quiescent population, and CD109, which is a marker for a more abundant proliferative progenitor cell type [20]. Both of these markers were coexpressed with the ∆Np63 isoform and demonstrated great proliferative capacity in vitro; however, only CD200-positive cells generated holoclones that are a hallmark of a self-renewing stem cell population in vitro. The more detailed knowledge of the mutual relations among various LSC markers and their exact positions in the functional LSC hierarchy have remained largely unknown.

In recent years, it has become increasingly evident that several stem cell niches in various tissues possess heterogenic stem cell subpopulations with distinct roles. For example, in the well-studied gut epithelium, LGR5-positive intestinal stem cells (ISCs) are located at the bottom of intestinal crypts together with nurturing Paneth cells, from where their short-lived transiently amplifying cell (TAC) progeny migrate towards the villi and terminally differentiate into mature intestinal epithelial cell types. On the other hand, it has been suggested that in the intestinal niche, there is also quiescent subpopulations of LGR5-negative ISCs, which upon injury activate to repopulate both the intestinal epithelium and the LGR5-positive cell pool, demonstrating the extreme flexibility of tissue repair mechanisms in the intestine [21]. It is currently not known whether corresponding stem cell compartments can also be found in the limbus. Intriguingly however, it has been shown that LSCs share some mutual markers with ISCs, such as LGR5 [22, 23] and BMI1 [11], suggesting potential stem cell compartmentalization in the limbal niche as well.

In this study, we utilized our established protocol [24, 25] to address the question of whether distinct stem cell populations can be identified during the in vitro differentiation process of hPSC-derived LSCs. Extensive characterization of the protein expression patterns during the differentiation process was carried using a set of putative stemness, LSC, and mature CE markers. With this approach, we revealed the subsequent emergence of three cell populations, each of which exhibited a different LSC-associated phenotype. The first two populations appeared early during the process and consisted mainly of cells with strong expression of ABCG2 but different levels of ∆Np63α. After this phase, the population phenotype gradually shifted to form the third population with strong expression of ∆Np63α and no ABCG2. By employing the Wnt/BMP signaling modifiers traditionally used for culturing ISCs, we were able to regulate the maintenance of ABCG2-positive hPSC-LSCs in vitro*.* Importantly, in the functional experiments, these ABCG2-positive hPSC-LSCs demonstrated increased regenerative potential in comparison to the cell population expressing the ∆Np63α-positive phenotype.

## Materials and methods

### Experimental design

**Fig. 1:**
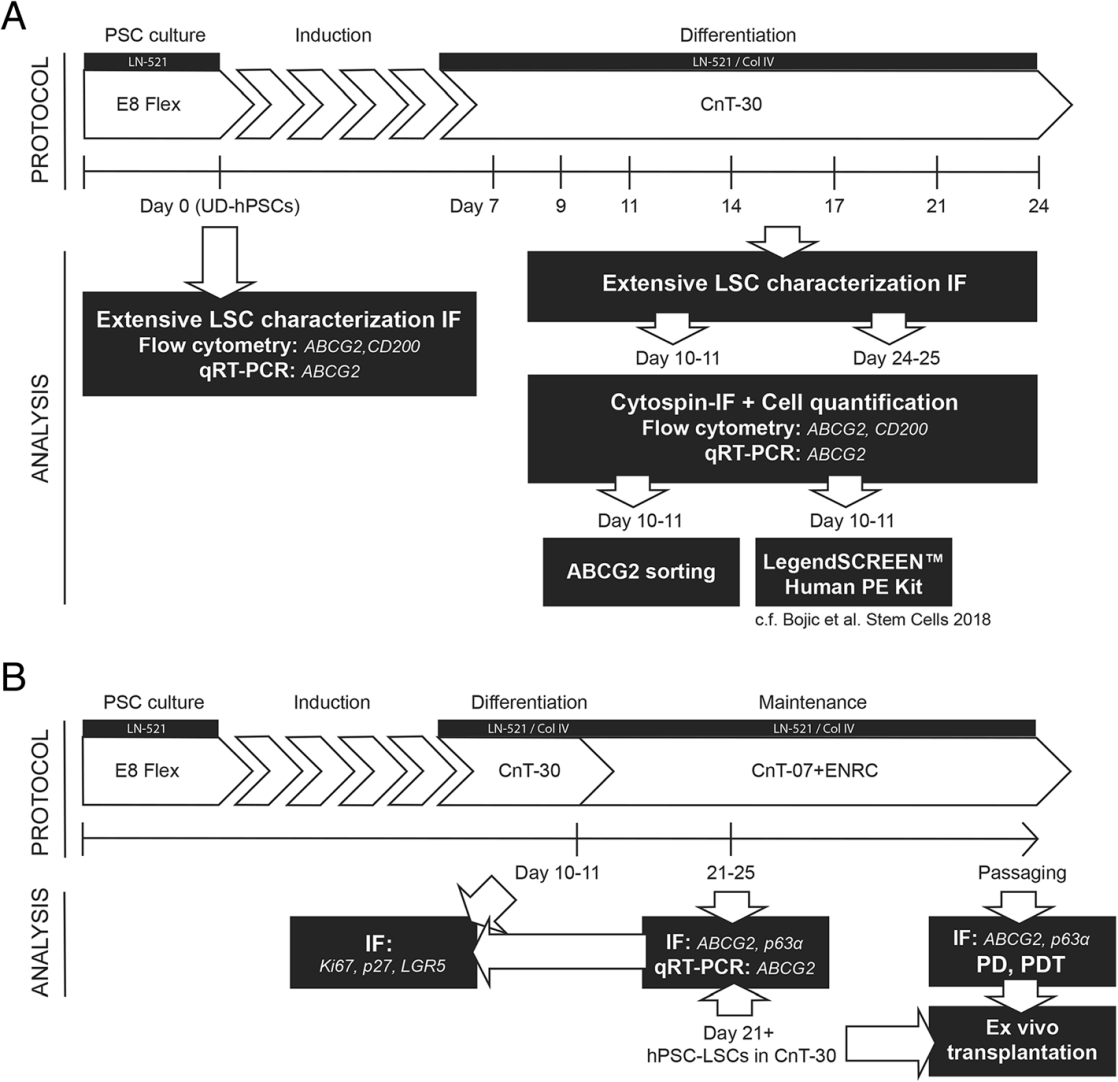
Flow chart of the experimental design and progression. **a** Standard CnT-30-based hPSC-LSC differentiation protocol and characterization of the hPSC-LSC differentiation process. **b** Novel CnT-07+ENRC-based hPSC-LSC maintenance protocol, characterization, and comparison of distinct cell populations identified during the study. PSC pluripotent stem cell, UD-hPSC undifferentiated human PSC, LSC limbal stem cell, IF immunofluorescence, qRT-PCR quantitative real-time PCR, LN-521 laminin-521, Col IV collagen type IV, E8 Flex, E8 Flex pluripotent stem cell culture medium, CnT-30 CnT-30 corneal differentiation medium, CnT-07 CnT-07 epithelial proliferation medium, ENRC epidermal growth factor, Noggin, R-Spondin-1, CHIR99021

Initial experimental design and progression of the study is presented in Fig. 1. The study consisted of two main parts, the first being the detailed characterization of hPSC differentiation process towards LSCs (Fig. 1a), and the second being establishing novel culture conditions for the maintenance of an ABCG2-positive LSC phenotype and further characterization of the stemness and functionality of the distinct populations observed in indicated time points and culture conditions (Fig. 1b). Full descriptions of the cell culture and cell characterization methods are provided as Supplemental Materials and Methods (Additional file 1).

### hPSC differentiation and hPSC-LSC culture

All three hPSC lines used in this study (hESC lines Regea08/017 and Regea11/013 and hiPSC line UTA.04607.WT) were derived and characterized in-house, as described previously [26, 27]. Human PSC cultures were routinely maintained in serum- and feeder cell-free conditions and differentiated towards the corneal epithelial lineage as described by Hongisto et al. [24, 25]. In brief, UD-hPSCs were enzymatically dissociated to a single-cell suspension and transferred onto low-attachment plates for induction. Formation of embryoid bodies (EBs) was supported by adding 5 μM blebbistatin (Sigma-Aldrich) to the defined XF-Ko-SR medium for 1 day. During the following 3 days, XF-Ko-SR was first supplemented with 10 μM SB-505124 and 50 ng/ml human basic fibroblast growth factor (bFGF; PeproTech Inc., Rocky Hill, NJ) for 1 day and with 25 ng/ml bone morphogenetic protein (BMP)-4 (PeproTech Inc.) for 2 days, to push the differentiation towards surface ectoderm. EBs were then transferred onto plates coated with 0.5 μg/cm^2^ recombinant laminin-521 (LN-521, Biolamina, Sweden) and 5 μg/cm^2^ human placental collagen Type IV (Col IV, Sigma-Aldrich) for adherent differentiation in defined commercial CnT-30 corneal differentiation medium (CELLnTEC Advanced Cell Systems AG, Bern, Switzerland). Cells were thereafter cultured in CnT-30, with medium changes three times per week until subjected to characterization analyses or further experimental settings. Throughout the article, the term “CnT-30 differentiation/condition” refers to the standard CnT-30-based differentiation protocol described in this chapter. Representative cell morphology during the differentiation was imaged with a Nikon Eclipse TE2000-S microscope equipped with a DS-Fi1 camera (Nikon Instruments, Amsterdam, Netherlands).

### Establishment of the ABCG2-positive hPSC-LSC culture

For maintenance of the ABCG2-positive population, CnT-30 was replaced with CnT-07 (CELLnTEC Advanced Cell Systems AG, Bern, Switzerland) supplemented with *ENRC* (50 ng/ml mouse recombinant epidermal growth factor (*E*GF, Invitrogen), 100 ng/ml mouse recombinant *N*oggin, 1 μg/ml human recombinant *R*-spondin (both from PeproTech), and 3 μM *C*HIR-99021 (Stemgent)) at d10–11 of the standard CnT-30 differentiation protocol. New medium was introduced directly to the adherent cultures; alternatively, the hPSC-LSCs were concomitantly passaged onto fresh LN-521/Col IV-coated wells at a density of 1 000 cells/cm^2^ in the new medium. The cells were thereafter cultured following the standard feeding regimen. After the emergence of ABCG2-positive colonies in approximately 7–10 days, further expansion of the ABCG2-positive hPSC-LSCs in the ENRC medium was carried out by passaging subconfluent cultures onto fresh LN-521/Col IV-coated matrices at a density of 1 000 cells/cm^2^. Batches of the passaged cells were also cryopreserved following our routine cryopreservation protocol [24, 25]. Throughout the article, the term “ENRC maintenance condition” refers to the novel CnT-07-based, ENRC-supplemented culture protocol described in this chapter.

### Immunofluorescence

Immunofluorescence staining (IF) was utilized in several stages during the study to analyze the protein expression of the cells, as presented in Fig. 1. In the characterization of standard hPSC-LSC differentiation in CnT-30, adherent cultures of UD-hPSCs as well as hPSC-LSCs at d7, d9, d11, d14, d17, d21, and d24 were stained with antibodies against OCT3/4, PAX6, ABCG2, ∆Np63, p63α, CK14, CK15, and CK12. Cytospin samples were prepared at d10 and d24 and stained with OCT3/4, ABCG2, p63α, ∆Np63, CK14, and CK15 for quantification of the different populations by cell counting analysis. Two hPSC lines (Regea08/017 and UTA.04607.WT) were used for the full IF characterization, and the results were replicated with at least two individual cell differentiation batches for both lines. Expression of indicated markers during standard differentiation of the third hPSC line, Regea11/013, was analyzed less extensively at d10–11 and d24–25.

Adherent d10–11 and d21–24 hPSC-LSCs during Cnt-30 differentiation as well as d21–24 hPSC-LSCs in ENRC maintenance were stained against ABCG2, p63α, LGR5, Ki67, and p27. Day 24 hPSC-LSCs in ENRC maintenance were characterized also for their OCT3/4, PAX6, CK14, and CK15 expression. Standard fixation and IF procedures with primary and secondary antibodies were performed essentially as described previously in Mikhailova et al. [28]. Raw images of the stained cells were captured with an Olympus IX51 fluorescence microscope. ImageJ Image Processing and Analysis tools [29] and Adobe Photoshop CC 2019 software were used for cell counting and image processing, respectively. Antibody specifics are provided as Supplemental Information (Additional file 2: Table S1).

### Flow cytometry and fluorescence-activated cell sorting

UD-hPSCs and d10–11 and d24-d29 hPSC-LSCs during Cnt-30 differentiation were characterized for their ABCG2 and CD200 surface antigen expression using flow cytometry. For practical reasons, cryopreserved d26–d29 hPSC-LSCs were used in some repeats in place of freshly differentiated cells, as we have previously demonstrated preservation of the phenotype throughout this process [24]. Standard flow cytometry staining protocols were used in the sample preparation, following the recommendations provided by the antibody manufacturers. APC-conjugated monoclonal mouse anti-human CD338 (ABCG2) antibody, clone 5D3 (BD Pharmingen, #561451), and PE-conjugated mouse monoclonal CD200 (clone OX-104) antibodies from two manufacturers (BioLegend, #329205 and BD Pharmingen, #561762) were used for indicated cell populations. An APC-conjugated mouse IgG2b κ antibody (BD Pharmingen, #555745) or unstained cells were used as isotype and/or negative controls, respectively. CD200-stained samples were also double-stained with ABCG2.

For continued culture of pure ABCG2-positive cell population, 1 000 Regea08/017 hPSC-LSCs staining positive for ABCG2 at d11 were sorted directly onto LN521/Col IV-coated wells in CnT-30 supplemented with a 10 μM concentration of the Rho kinase inhibitor Y-27632 (Tocris Bioscience) and were then cultured following the standard feeding regimen for 17 days. The sorted cells were stained in IF against p63α and ABCG2, and expression of p63α was quantified by cell counting.

Both flow cytometry analyses and cell sorting were performed using a BD FACSAria™ Fusion cell sorter operating with the FACSDiva™ software (BD Biosciences, San Jose, California, USA). At least 10 000 events were recorded from the initially gated populations and analyzed with FlowJo 10 software (BD Biosciences, San Jose, CA, USA).

### Quantitative RT-PCR

UD-hPSCs and d10–11 and d21–24 hPSC-LSCs during Cnt-30 differentiation as well as d21 hPSC-LSCs in the ENRC maintenance were analyzed for their ABCG2 mRNA expression with qPCR, using a sequence-specific TaqMan Gene Expression Assay for ABCG2 (#HS01053790_m1, Applied Biosystems). GAPDH (Hs99999905_m1) was used as a housekeeping gene. Standard methods were used for RNA isolation and cDNA synthesis from the cell pellet samples collected in indicated time points. All samples and controls were run as triplicate reactions with the 7300 Real-Time PCR system (Applied Biosystems). The results were analyzed using the − 2^∆∆Ct^ method [30] and are presented as the fold change in gene expression normalized to GAPDH and relative to the UD controls.

### Cell surface antigen screening

Cell surface marker screening was performed for the Regea08/017 hPSC-LSCs at d10 during Cnt-30 differentiation, using the LEGENDScreen™ Lyophilized Antibody Array, Human PE Kit (BioLegend, #700007) and essentially following the instructions of the manufacturer. The experiment was carried out in four parts using 120 000–150 000 cells per sample that were double-stained with 3 μl of APC-conjugated ABCG2 antibody (BD Pharmingen, #561451). At least 10 000 initially gated events per sample were recorded with FACSCanto II flow cytometer (BD Biosciences, San Jose, CA, USA) and analyzed with FlowJo software. The results were compared to the previously published screening data of cultured primary human LSCs, produced by Bojic et al. [20], with special emphasis on the two novel LSC markers, CD109 and CD200.

### Population doubling analyses

Population doubling calculations were carried out for the Regea11/013 hPSC-LSCs cultured in ENRC, both prior and after the standard cryopreservation protocols described in Hongisto et al. [24]. Population doublings (PDs) at the end of each subculture were calculated using the following formula: log (*N*/N0)/log2, where N0 is the number of plated cells and *N* is the number of cells at the end of the culture period. Similarly, the population doubling time (PDT) for each passage was calculated with the following formula: *T* × log2/log(*N*−N0), where *T* is the duration of the culture in hours.

### Ex vivo transplantation into a porcine cornea model

Porcine corneas were obtained and processed for corneal ex vivo culture as previously described in [31, 32]. The excised corneas were maintained in CnT-CC medium (CELLnTEC Advanced Cell Systems AG, Bern, Switzerland) for up to 3 weeks prior to transplantation experiments. LSCD mimicking state was induced to the ex vivo corneas by placing a filter paper disc soaked with 1 M sodium hydroxide (NaOH) onto the corneal surface for 40 s, followed by thorough removal of the epithelium by scraping.

Human PSC-LSCs cultured in CnT-30 and ENRC conditions were seeded onto both sides of a fibrin carrier membranes at a density of 30 000 cells/cm^2^ and thereafter cultured for 2 additional weeks in their initial medium conditions. The carrier membranes were transplanted into the ex vivo corneas using four interrupted 9-0 Vicryl sutures. Soft contact lenses were placed on top of the corneas to prevent drying. After ex vivo transplantation, the hPSC-LSCs from CnT-30 conditions were further cultured in CnT-30 for 1 week (*n* = 2). The hPSC-LSCs from ENRC conditions were cultured in CnT-07 for 1 week (*n* = 2) or CnT-30 for 2 weeks (*n* = 3). The medium was gradually changed from CnT-07 + ENRC to CnT-30 through one intermediate step with 1:1 ratio of the two media. During ex vivo culturing, all media were supplemented with 5% fetal bovine serum (FBS, Sigma-Aldrich), 1% penicillin/streptomycin and 0.1% amphotericin B (Sigma-Aldrich). Media were replaced three times a week. After 1 to 2 weeks, the ex vivo corneas were fixed, processed into paraffin-embedded tissue sections, and stained with hematoxylin-eosin (HE), following the standard methods. Images of the HE-stained tissue sections were captured with the Nikon Eclipse TE2000-S microscope and DS-Fi1 camera.

### Statistical methods

All data are presented as the mean ± standard deviation (SD). Whenever *n* ≥ 3, the Mann-Whitney *U* test was performed to analyze the differences between the groups using the GraphPad Prism 5 software (GraphPad Software Inc.). Differences were considered statistically significant when *P* ≤ 0.05.

## Results

### Dissection of the hPSC-LSC differentiation hierarchy reveals the subsequent emergence of three separate cell populations, marked by distinct expression patterns for ABCG2 and ∆Np63α

**Fig. 2:**
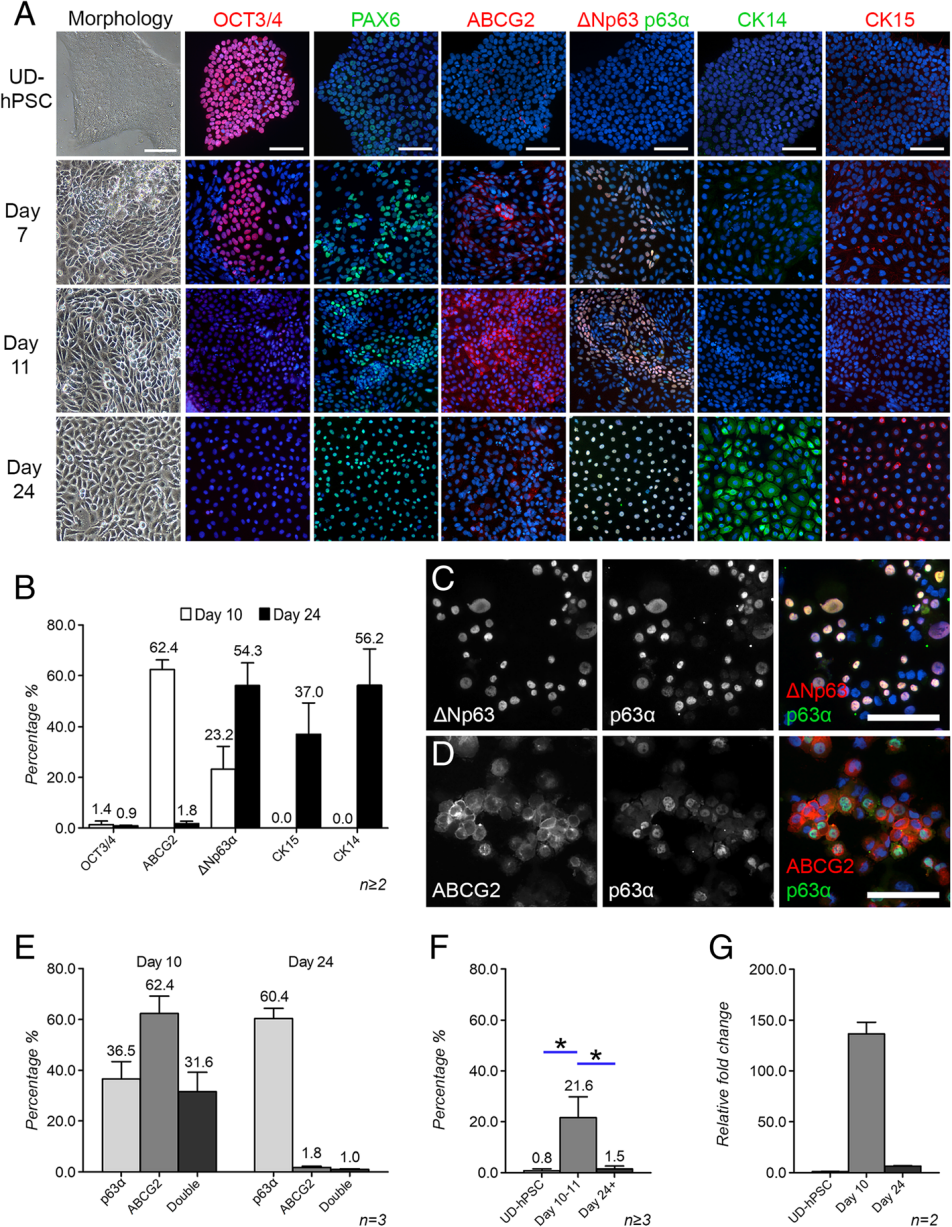
Characterization of putative LSC marker expression during hPSC-LSC differentiation. **a** Representative morphology and protein expression of the cultures at selected time points. Scale bars, 100 μm for all images in the same column. Cell nuclei counterstained with DAPI (blue). **b** Marker expression differences in the d10 and d24 populations. Five images per sample and a minimum of 1400 cells per time point were analyzed for each marker from cytospin samples. **c** Representative IF image of ∆Np63 and p63α double-staining in a d24 cytospin sample. Scale bar, 100 μm for both **c** and **d**. **d** Representative IF image of ABCG2 and p63α double-staining in a d10 cytospin sample. **e** p63α and ABCG2 expression in d10 and d24 hPSC-LSCs. Five images per sample and a minimum of 3 000 cells per time point were analyzed from cytospin samples. *P* > 0.05. **f** The level of ABCG2 protein expression in UD-hPSCs and in d10 and d24–26 hPSC-LSCs, analyzed with flow cytometry. **g** The ABCG2 mRNA expression levels in UD-hPSCs and in d10 and d24 hPSC-LSCs analyzed with qRT-PCR. All representative data are presented with the hESC line Regea08/017. All quantitative data are presented as the mean + SD, and *n* marks the individual cell differentiation batches serving as biological replicates. Statistical analyses were carried out using the Mann-Whitney *U* test. *P ≤ 0.05

To investigate protein expression patterns during the differentiation of hPSCs towards LSCs, UD-hPSCs were differentiated towards the corneal lineage and characterized using IF. During the time frame ranging from d7 to d24, expression of OCT-3/4 was markedly downregulated, whereas expression of PAX6, ∆Np63α, CK15, and CK14 increased, indicating the emergence of an LSC-like population (Fig. 2a). Interestingly, ABCG2 was expressed only transiently, peaking between d9 and d11 and then gradually decreasing to very low levels by d24 (Fig. 2a). In accordance with our previous results [24], CK12 remained undetectable within this timeframe (data not shown). Quantification of cell populations by protein expression confirmed major differences between the d10 and d24 time points, as shown for the representative hESC line (Regea08/017) in Fig. 2b. To be precise, the expression of ABCG2 decreased from 62.3% (SD 6.7) to 1.8% (SD 0.9), while the expression of ∆Np63α (as demonstrated by double-staining with ∆Np63 and p63α antibodies, Fig. 2c) increased from 23.2% (SD 14.1) to 54.3% (SD 6.2). CK15 and CK14 were undetectable at d10 but increased to 37.0% (SD 12.4) and 56.2% (14.3) by d24, respectively. OCT3/4, on the other hand, was expressed in less than 1.5% of the cells at d10 and was further diminished to under 1% by d24. Due to the distinct expression profiles of ABCG2 and ∆Np63α during the differentiation process, we further characterized the coexpression of ABCG2 with p63α in d10 and d24. Interestingly, the most intense expression of both markers was typically observed in separate cell populations, as demonstrated in Fig. 2d (see also Additional file 3: Figure S1), suggesting a transitional rather than a stable phase for the coexpression. At d10, ABCG2 and p63α were coexpressed in 31.6% of the cells, whereas at d24 only 1% of the cells were double-positive (Fig. 2e).

Flow cytometry confirmed the IF results by showing that both UD-hPSCs and more differentiated d24–26 hPSC-LSCs had low expression of ABCG2 (0.8%, SD 1.3 and 1.5%, SD 2.0, respectively, for the representative hESC line Regea08/017), whereas d10–11 hPSC-LSCs expressed significantly higher levels of ABCG2 (21.6%, SD 8.2) than both UD-hPSCs (*P* = 0.0238) and d24–26 hPSC-LSCs (*P* = 0.0275) (Fig. 2f, see also Additional file 4: Figure S2A for representative flow cytometry plots). Lower percentage of ABCG2 expressing cells in flow cytometry in comparison to cytospin quantification is likely in large part a technical issue related to the chosen methods, as standard IF sample processing (including permeabilization) also allows staining of the intracellular ABCG2, resulting in more abundant positive signal, whereas in live cell flow cytometry only the cells expressing ABCG2 on their surface membrane label as positive.

Notably, isolation of ABCG2-positive hPSC-LSCs at d11 leads to the formation of homogeneous hPSC-LSC monolayers, with 99.9% (SD 0.2, *n* = 2800 cells) nuclear p63α expression and very low to undetectable levels of ABCG2 (Additional file 4: Figure S2B). Additionally, changes in ABCG2 expression at the mRNA level further verified notably higher expression level of ABCG2 in the d10 hPSC-LSC population than in UD-hPSCs and the d24 hPSC-LSC population (Fig. 2g). Taken together, the characterization analyses consistently demonstrated very distinct expression profiles for the proposed LSC/progenitor markers ABCG2, ∆Np63α, CK15, and CK14 between the d10–11 and d24 time points during the hPSC-LSC differentiation. Importantly, the described expression patterns were reproduced with the hiPSC line UTA.04607.WT (Additional file 5: Figure S3).

### Screening of LSC-associated surface markers revealed a high level of CD200-positive cells among d10 hPSC-LSCs

At d10 in Cnt-30 culture, Regea08/017 hPSC-LSCs expressed several limbus-associated markers at levels comparable to those of human primary cultured LSCs, as reported by Bojic et al. [20] (Table 1). Furthermore, we specifically investigated the two markers highlighted by Bojic et al. as novel candidates for quiescent LSCs and the proliferative progenitor phenotype, namely, CD200 and CD109. CD200 was expressed at a considerably higher level in our d10 hPSC-LSCs than in cultured primary human LSCs (42.6% vs. 2.3%, respectively). On the other hand, CD109 was expressed in only 25.7% of hPSC-LSCs, in comparison to 56.3% of primary cultured LSCs. Interestingly, ABCG2 was expressed in approximately half of both the CD200- and CD109-positive hPSC-LSC subpopulations, thus showing no preferred coexpression with either of these markers. To be precise, there were 48.1% ABCG2-positive and 51.9% ABCG2-negative cells in the CD200-positive population, and in the CD109-positive population, these numbers were 58.5% and 41.5%, respectively (Additional file 6: Table S2).

**Table 1:** Expression of selected cell surface markers in d10 Regea08/017 hESC-LSCs

Surface marker	hPSC-LSCs (%)	Primary human LSCs [20] (%)
EGFR	97.7	88.8
CD71	81.9	88.8
Integrin β5	93.5	91.5
Integrin α6 (CD49f)	98.6	92.5
E-cadherin (CD324)	85.9	88.5
CD40	24.7	26.0
CD146	84.0	67.0
CD166	99.5	95.1
CD200	42.6	2.3
CD109	25.7	56.3

**Fig. 3:**
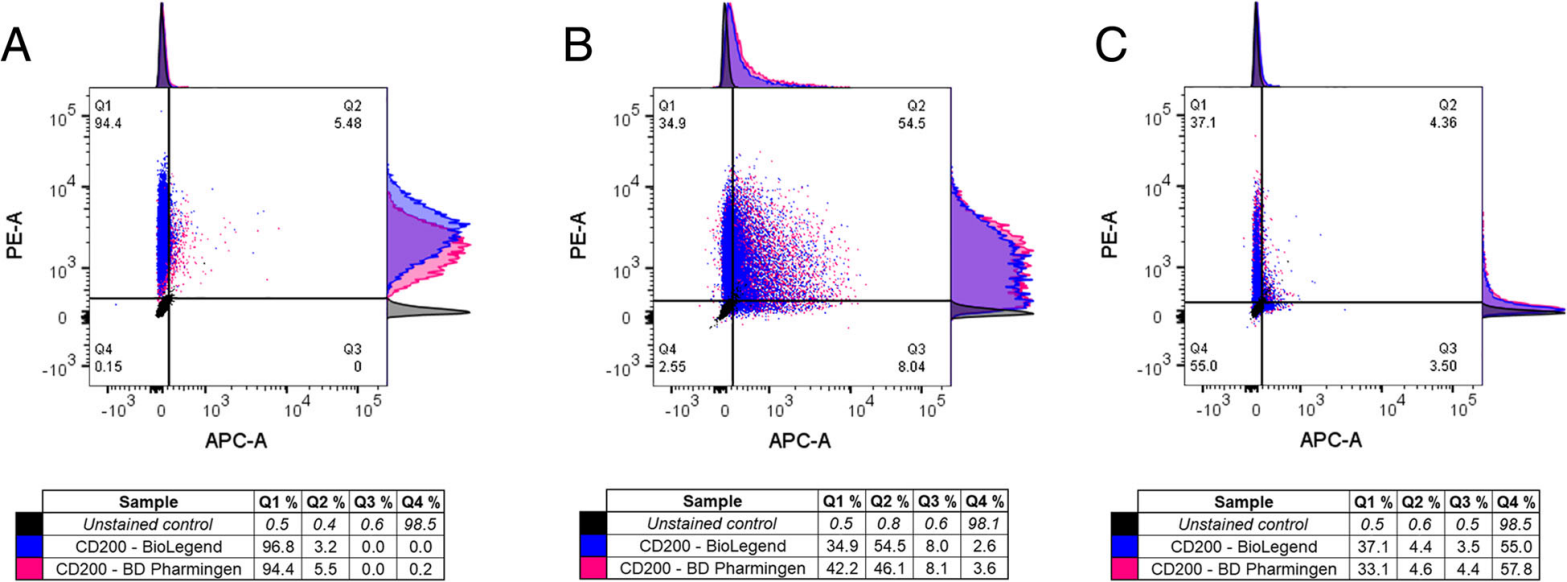
CD200 and ABCG2 expression patterns during differentiation of hPSC towards LSCs, analyzed with flow cytometry. Scatter plots and adjunct histograms as well as tables showing the distribution of cells in the CD200^+^/ABCG2^−^ (Q1), CD200^+^/ABCG2^+^ (Q2), CD200^−^/ABCG2^+^ (Q3), and CD200^−^/ABCG2^−^ (Q4) subpopulations of UD-hPSCs (**a**) as well as at d11 (**b**) and d29 (**c**) during hPSC-LSC differentiation. For each sample, 10,000 initially gated events were analyzed, and the experiment was carried out once for the representative line Regea08/017

As the role of CD200 was recently investigated also in hPSC-derived corneal cells [33], we performed flow cytometry analysis to further analyze the expression pattern of CD200 during differentiation of hPSC-LSCs. The results were consistent with both used CD200 antibodies, unambiguously showing that CD200 was expressed in over 99% of UD-hPSCs (Fig. 3a). During the Cnt-30 differentiation, CD200 expression decreased from 87.6% (SD 0.7) at d11 to 38.5% by d29 (Fig. 3b, c), whereas ABCG2 expression followed a typical trend, with low expression in 3.3% (SD 1.8) of UD-hPSCs, 57% (SD 5.9) of d11 cells, and 7.4% (SD 0.9) of d29 cells (Fig. 3a–c). Again, based on our results, the expression pattern of CD200 did not correlate with that of ABCG2, and in line with the screening results, there were both ABCG2-positive and ABCG2-negative hPSC-LSCs in the CD200-positive subpopulation. However, we were unable to confirm this specific finding with IF visualization using the unconjugated primary CD200-antibody from BioLegend.

### Maintenance of ABCG2 expression during hPSC-LSC culture is achieved with Wnt/BMP pathway signaling modifiers previously established for the long-term sustained growth of intestinal organoids in vitro

**Fig. 4:**
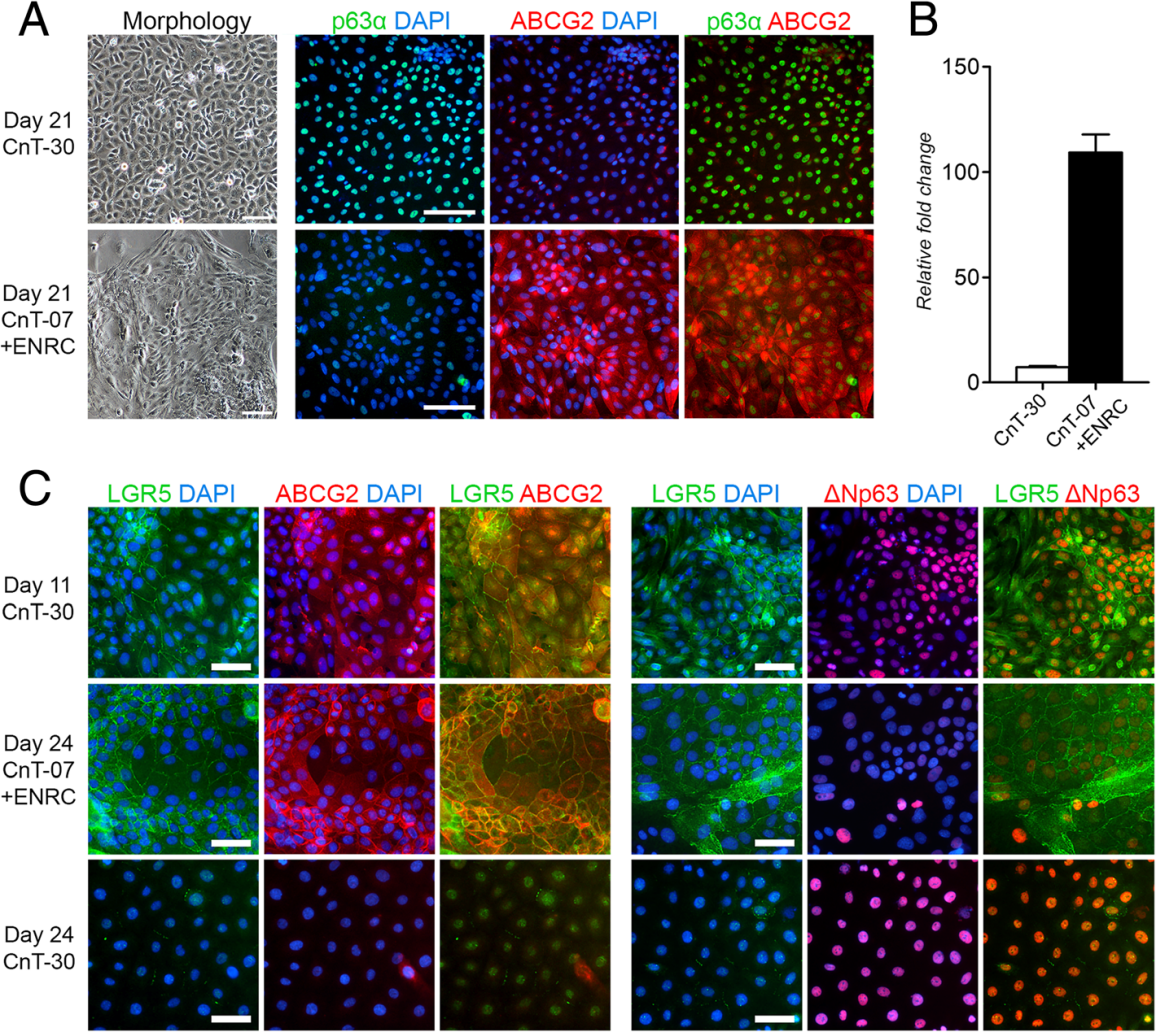
Effect of culture conditions on hPSC-LSC morphology and p63α, ABCG2, and LGR5 expression**. a** Representative cell morphology and p63α/ABCG2 protein expression under different culture conditions, as demonstrated by IF. Scale bars, 100 μm. **b** ABCG2 mRNA expression under different conditions, analyzed with qRT-PCR in d21 cells. Data are presented as the mean + SD, *n* = 3 technical replicates from one sample, *P* > 0.05. **c** Characterization of LGR5 protein expression in relation to ABCG2 and ∆Np63 at d11, as well as after continued culture in CnT-07+ENRC or CnT-30 at d24. Cell nuclei counterstained with DAPI (blue). Scale bars, 50 μm. Data shown for the representative hESC line Regea08/017

To study the properties of the ABCG2-positive cell population in more detail, culture conditions aiming to preserve the ABCG2 expressing cell population for extended periods were established. Due to similarities between the limbal and intestinal crypt stem cell niches and the signaling pathways involved in the regulation of stemness, we hypothesized that Wnt agonists and a BMP antagonist previously used for intestinal crypt organoid culture could be utilized for regulating the differentiation process of hPSC-LSCs as well. Remarkably, replacing the corneal differentiation medium CnT-30 with the epithelial maintenance medium CnT-07 and specific combination of EGF, Noggin, R-spondin-1, and CHIR-99201 (ENRC) at day 11 of differentiation, resulted in the preservation of the colonial morphology and strong ABCG2 protein expression that were observed only transiently during the CnT-30 differentiation. The main differences between the standard CnT-30-based differentiation condition and the novel CnT-07+ENRC-based maintenance condition are shown in Fig. 1. Notably, colonies positive for ABCG2 in the ENRC condition expressed only low levels of p63α, whereas time point-matched cells in CnT-30 were ABCG2-negative and p63α-“bright” (Fig. 4a). These IF results were confirmed with all three studied hPSC lines. Importantly, the effect of the ENRC condition was consistent in all lines, despite cell line-specific variations in the efficacy. Prominent upregulation of ABCG2 at the mRNA level in the ENRC condition in comparison to the CnT-30 condition was confirmed also with qRT-PCR using the hESC line Regea08/017 (Fig. 4b). Additional characterization of PAX6, CK14, CK15, and OCT3/4 expression in Regea11/013 hPSC-LSCs in the maintenance condition demonstrated positive expression for PAX6, weak expression for CK14 and CK15, and negative expression for OCT3/4 (Additional file 7: Figure S4A).

### ABCG2-positive limbal colonies coexpress the stem cell marker LGR5

Due to the finding that ENRC supplementation, which preserves LGR5-positive ISCs, also supports the maintenance of the ABCG2-positive LSC phenotype, we decided to analyze the expression of LGR5 in our cells and compare its expression pattern to those of both ABCG2 and ∆Np63. Indeed, prevalent expression of LGR5 was observed during CnT-30 culture at d11, followed by decreased expression upon further differentiation up to d24. On the other hand, in time point-matched colonies in ENRC, the expression of LGR5 was highly conserved (Fig. 4c). Interestingly, at d11 during the CnT-30 culture, LGR5 was coexpressed with ∆Np63, as marked by distinct membrane-localized staining at cellular junctions of ∆Np63-positive cells (Fig. 4c). Further differentiation under CnT-30 conditions, resulting in the formation of a ∆Np63-positive epithelial monolayer, was accompanied by concomitant loss of strong LGR5 expression on the cell surface, a phenomenon similar to the expression pattern of ABCG2. Under ENRC conditions, LGR5 expression was localized to cellular junctions, again similar to ABCG2. Fascinatingly, as in the ENRC, the colonies expressed only low levels of ∆Np63; LGR5 thus appeared to switch its preferred coexpression with ABCG2 and ∆Np63, depending on the culture conditions.

### ABCG2-positive hPSC-LSCs retain clonal growth and proliferative capacity during passaging in the novel maintenance condition

**Fig. 5:**
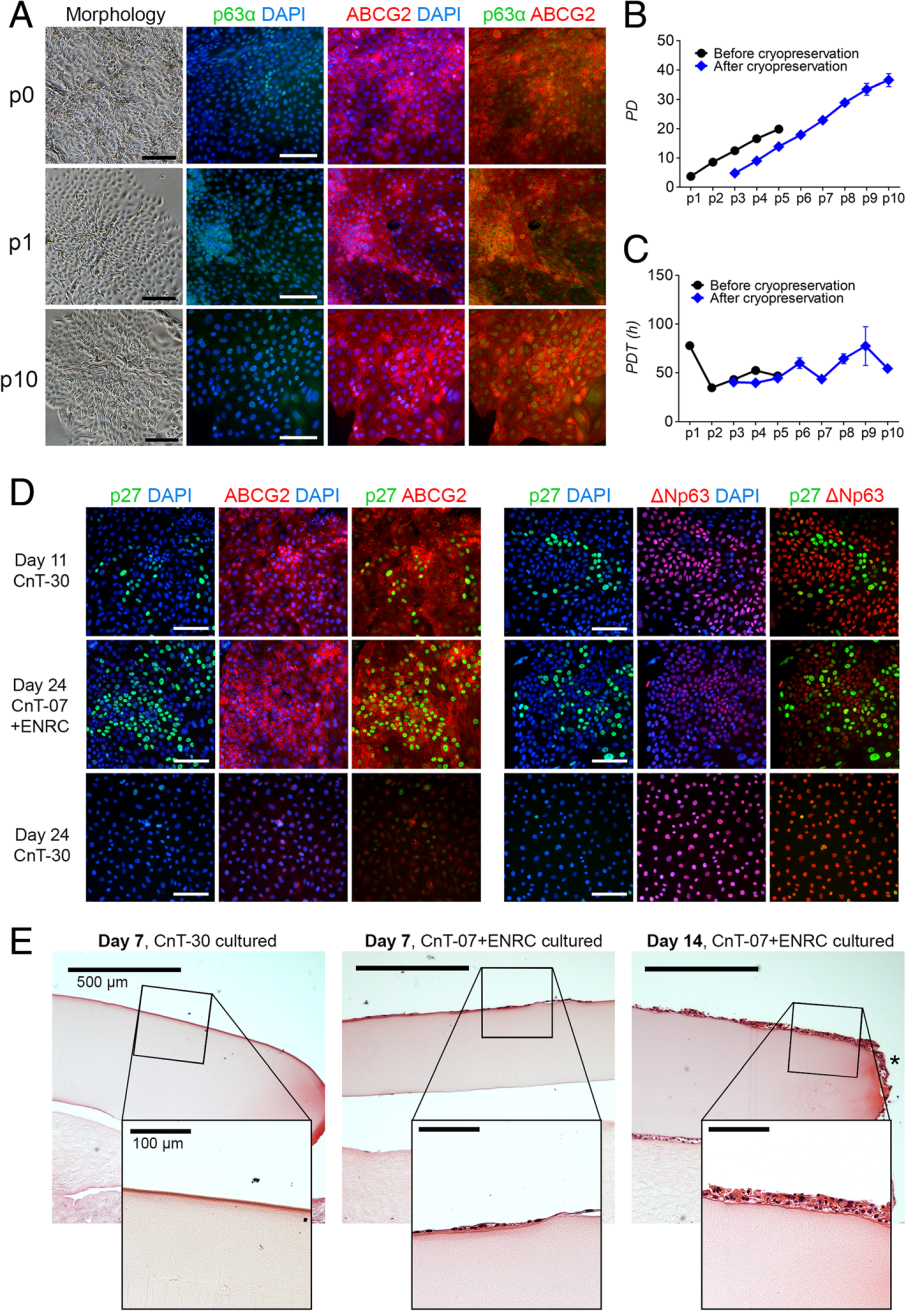
Marker expression and functional proliferative and regenerative properties of the ABCG2-positive hPSC-LSCs cultured in the novel maintenance condition**. a** Morphology and p63α/ABCG2 expression pattern of hPSC-LSCs cultured in CnT-07+ENRC up to passages 0, 1, and 10. Cell nuclei counterstained with DAPI (blue). Scale bars, black = 200 μm, white = 100 μm. **b** Population doublings and **c** population doubling times of freshly differentiated hPSC-LSCs up to passage 5 and cryopreserved hPSC-LSCs up to passage 10. **d** Characterization of expression of the quiescence marker p27 in relation to ABCG2 and ∆Np63 at d11, as well as after continued culture in CnT-07+ENRC or CnT-30 at d24. Scale bars, 100 μm. **e** Hematoxylin and eosin-stained sections of CnT-30-cultured Regea08/017 and CnT-07+ENRC-cultured Regea11/013 hPSC-LSCs after 1-week and 2-week time points in the ex vivo porcine cornea model, respectively. Asterisk points out migrating cells. Scale bars, 500 μm and 100 μm, for all images in the panel. Data are presented for the hESC lines Regea11/013 (**a**–**c**, **e**) and Regea08/017 (**d**, **e**)

Population doubling analyses were carried out to determine the proliferative capacity of hPSC-LSCs. Under ENRC maintenance, colony morphology and the ABCG2/p63α expression pattern were preserved at least up to passage 10 (Fig. 5a). As comparison, passaging and subsequent culturing in CnT-30 medium resulted in loss of the colony morphology and ABCG2 expression and promoted further differentiation towards ∆Np63α-positive epithelial monolayers, as described for the hPSC-LSC differentiation process (shown in, e.g., Fig. 4a). In addition, the proliferation of hPSC-LSCs is rapidly diminished upon passaging in CnT-30 and generally ceases after the third passage, as repeatedly demonstrated with various cell lines during our standard hPSC-LSC cell culture routine.

To quantify the proliferative capacity of the cells, Regea11/013 hPSC-derived LSCs were frequently passaged under ENRC conditions, and their PDs and PDTs were calculated. Freshly differentiated Regea11/013 hPSC-LSCs were cultured for five passages, during which the cells went through over 20 population doublings, with an average PDT of 50.9 h (SD 16.4) (Fig. 5b, c, black line). Notably, cryopreservation between passages 2 and 3 (p2-p3) did not have a marked effect on the proliferative capacity of the cells after thawing (Fig. 5b, c, blue line). After cryopreservation, the average PDT of Regea11/013 hPSC-LSCs was 52.9 h (SD 13.5), which was not significantly different than the PDT of freshly differentiated cells prior to cryopreservation (unpaired *T* test, *P* = 0.8086). In the course of eight passages (from p3 to p10) under ENRC, cryopreserved Regea11/013 hPSC-LSCs went through 36.6 population doublings (Fig. 5b, blue line) without evident signs of cellular senescence, as indicated by rather stable PDTs (Fig. 5c, blue line).

### ABCG2-positive hPSC-LSC colonies contain a subpopulation of quiescent stem cells and demonstrate regenerative potential in an ex vivo porcine cornea model

We further investigated the proliferative status of the distinct stem cell populations that emerged during CnT-30 differentiation as well as under the ENRC condition, by characterizing their expression of the proliferation marker Ki67 and the quiescence-related marker p27. Not surprisingly, Ki67 stained a large subpopulation of cells during CnT-30 differentiation as well as in the ABCG2-positive, clonally growing colonies in the ENRC condition (Additional file 7: Figure S4). The ABCG2-negative, presumably ∆Np63α-positive population at the later time point also contained a large subpopulation of Ki67-positive cells (Additional file 7: Figure S4B).

Interestingly, ABCG2-positive colonies under the ENRC condition as well as at d11 during CnT-30 differentiation contained subpopulations of quiescent cells marked by strong nuclear localization of p27, whereas at later time points in the CnT-30 culture, p27 expression was markedly decreased. ∆Np63 and p27 expressions were mutually exclusive in all the studied conditions (Fig. 5d). Importantly, when transplanted into an ex vivo porcine cornea model, the ENRC-cultured ABCG2-positive hPSC-LSCs produced a viable multilayered epithelium with robust wound healing capacity, as indicated by cells that had migrated to the sides of the carrier gel at 2-week time point (Fig. 5e, on the right). A monolayer of ENRC-cultured cells was observed also at 1-week time point (Fig. 5e, in the middle) whereas no CnT-30-cultured ∆Np63α-positive hPSC-LSCs were observed to be left in the ex vivo cornea cultures after this time (Fig. 5e, on the left).

## Discussion

Extensive research on the CE has established a solid base for understanding the general aspects of its renewal. However, further knowledge of the LSC population identity as well as the regulation of LSC maintenance and activation in the human cornea is required to produce more efficient and safer methods for clinical purposes. One of the key challenges in developing hPSC-based treatments has been the production and isolation of cell material that robustly exhibits essential hallmarks of adult LSCs and lacks the tumorigenic properties of PSCs. This task is even more challenging because the identity of the LSCs is still under debate. In this paper, we describe a detailed phenotypic characterization of hPSC-derived LSCs during differentiation and propose causal relationships between acknowledged and novel LSC markers. Our results suggest the existence of separate stem cell populations within hPSC-LSCs that are similar to those that have been previously identified, for example, in the bone marrow, skin, and intestine [34]. Furthermore, in our study, the in vitro maintenance of the cell population with LSC characteristics was achieved by modulating the Wnt and BMP pathways, mimicking the maintenance conditions commonly used for ISCs.

∆Np63α is one of the most widely used LSC markers. It has been reported that 10% of resting limbal basal cells show nuclear ∆Np63α staining with stem cell properties. A fraction of these cells have been shown to become activated upon injury and acquire a proliferative ∆Np63α-positive phenotype [11]. Upon activation, LSCs gradually lose their self-renewal properties along with ∆Np63α expression and convert to TACs with limited proliferation potential [8, 11]. In our culture environment, changes in the marker expression profile following small-molecule induction and CnT-30-based standard differentiation of hPSCs indicated a shift from pluripotency towards the corneal epithelial lineage, as reported previously by our group [24, 25, 28]. After being cultured in CnT-30 conditions for 24 days, over 50% of the cells expressed nuclear ∆Np63α, however, raising the question of whether these cells still represented LSCs or had already started to commit towards early TACs. Interestingly, dissection of the developmental trajectory revealed a transient expression phase (d9–11) of another potential LSC marker, ABCG2. The membrane localization of ABCG2, an efflux transporter, in these cells indicated that this protein was able to attain a functional conformation [35]. Surprisingly, the spatial expression of p63 and ABCG2 both in vivo and in vitro has been poorly addressed in the literature. In our study, coexpression of ABCG2 and ∆Np63α upon early CnT-30 differentiation was limited to under 32% of the cells and was reduced to only 1% of the cells at d24, indicating a transitional rather than coexpressional status of these markers. Furthermore, the cells with robust ABCG2 expression and membrane localization had relatively “dim” nuclear ∆Np63α staining, whereas ∆Np63α-“bright” cells that appeared in the culture upon further differentiation typically did not express ABCG2. When selecting only the ABCG2-positive cells for continued culture in the CnT-30 differentiation medium, robust generation of a pure ABCG2-negative and p63α-“bright” epithelial monolayer was observed, indicating that these cells originate from same founder but undergo rapid early-stage lineage commitment in the hPSC-LSC differentiation pathway.

To date, some discrepancy remains concerning whether there is one slow-cycling LSC population that is also able to self-renew [11] or two separate LSC populations, of which one is fully quiescent and the other represents activated but slow-cycling LSCs that can produce progeny for quiescence, self-renewal, and differentiation [36]. To further elucidate the identity of the hPSC-derived LSCs within the time-window showing ABCG2 expression, we carried out an extensive screening analysis to compare our results to those previously acquired with human primary LSCs by Bojic et al. [20]. Based on this comparison, many limbal proteins were expressed similarly between hPSC-LSCs and primary cells, apart from CD200 and CD109, novel cell surface markers of putative quiescent and active LSCs, respectively [20]. In our hPSC-LSC cultures at d10, the number of CD200-positive cells was much higher, which could potentially be explained by the wide range of different cell types known to express this marker. In fact, Hayashi et al. used CD200 as a negative selector for hiPSC-derived corneal epithelial cells and reported a lack of a CD200 signal in adult human and mouse corneas [33]. However, in their study, the selection was performed after 10–15 weeks of differentiation, at which stage the remaining CD200-positive population may already be committed to lineages other than the corneal lineage. On the other hand, the lack of a signal from the corneal tissue in the study by Hayashi et al. may be affected by the time from death to retrieval of tissue, which was not reported but should be kept short to avoid the loss of stem cells. In our study, it is notable that almost all of the ABCG2-positive cells were also positive for CD200, indicating that a subpopulation of CD200-expressing cells may also be LSC-like in the hPSC-derived cultures. On the other hand, CD109 was expressed in fewer hPSC-LSCs than primary LSCs [20], possibly reflecting the rapid differentiation pace and potential commitment of hPSC-LSCs towards the TAC phenotype in CnT-30. Primary cell cultures also typically contain nurturing limbal niche cells that support the quiescence of LSCs [36, 37]. This environment is mimicked only partially in our cell cultures by chemical and ECM signals, which could drive all or some of the hPSC-LSCs into an activated state, as suggested by the expression of the proliferation marker Ki67 by a large number of the cells, including many of the ABCG2-positive cells. Regardless, it seems that a subpopulation of the ABCG2-positive cells is actually quiescent in our hPSC-LSC cultures based on immunostaining of p27, a reversible cell cycle arrest marker [38]. Whether these quiescent cells were also positive for CD200 warrants further study with functional antibodies. In vivo, quiescence in the human limbus has been linked to ∆Np63α positivity based on coexpression of ∆Np63α and p27 [11]. The p27 protein belongs to the Cip/Kip family of cyclin-dependent kinase (CDK) inhibitor proteins that prevent the activation of the cyclin E-CDK2 and cyclin D-CDK4 complexes and control cell cycle progression at G1, resulting in slower or arrested cell division. Importantly, to inhibit CDK, p27 needs to be localized in the nucleus [39, 40]. In the study by Barbaro et al., IF from human limbal tissue showed that p27 localized predominantly in the cytoplasm and only weakly in the nucleus [11], suggesting that the ∆Np63α/p27-expressing cells may have been exiting the quiescent phase and/or represented slow-cycling cells. In our study, p27 staining in cell cultures was predominantly nuclear, suggesting full cell cycle arrest.

Wnt signals are an essential component of a wide range of stem cell niches, including the limbus and the intestine, and interestingly, renewal of the CE highly resembles the regeneration of the gastrointestinal epithelium. ISCs are located in the bottom of intestinal crypts, from where their short-lived TA progeny migrate towards the villi and terminally differentiate into mature intestinal epithelial cell types. The differentiation mechanisms of ISCs employing the Wnt/BMP regulatory routes are currently well understood and have been successfully applied to the ex vivo*/*in vitro study of ISCs [41]. There are also studies suggesting similar regulation of quiescence and stem cell activation/renewal by the Wnt/BMP signaling pathways in the limbus. In general, Wnt signaling and suppression of BMP are required for LSC proliferation and maintenance of an uncommitted state [42, 43]. However, the outcome appears to be dependent on the culture system used, as the same exogenous signals have been reported to have opposite effects in explant versus isolated limbal cultures [44]. In our study, we tested the effect of the ENRC growth factor cocktail that activates Wnt signaling and inhibits BMP and is commonly used for long-term ex vivo culture of ISCs [41] on the differentiation and population maintenance of hPSC-LSCs. Together with commercial CnT-07 medium specifically designed for epithelial proliferation instead of the corneal differentiation medium CnT-30, ENRC supplementation led to prolonged maintenance of ABCG2-positive cells. Similar to the CnT-30 differentiation culture at d11, the ENRC-cultured hPSC-LSCs expressed PAX6 and stained faintly positive for the basal epithelial cytokeratins 14 and 15, and the ABCG2-positive colonies were ∆Np63α-“dim” and contained a subpopulation of p27-positive/∆Np63α-negative cells. However, unlike the CnT-30 cultured ABCG2-negative/∆Np63α-“bright” populations, ENRC maintained colonial growth of the cells until at least passage 10 without signs of cellular senescence, during which time the cells underwent 37 population doublings. Additionally, we found that LGR5, an established marker for ISCs [45] and a suggested marker for LSCs [22, 23], showed spatiotemporal coexpression with ABCG2 in both CnT-30 cultures at d11 and ENRC cultures. It is now commonly acknowledged that there are two populations of ISCs with distinct roles. The LGR5-marked stem cells reside in the crypt bottom adjacent to the nurturing Paneth cells and are proliferative yet long-lived, maintaining a rather undifferentiated phenotype. These cells are the main population responsible for producing the differentiating TAC progeny under homeostatic conditions. On the other hand, the quiescent cells in the + 4 position do not contribute to homeostatic regeneration but activate upon injury to both repopulate the intestinal epithelium and depleted LGR5-positive ISC pool. This process can also act vice versa, and LGR5-positive cells can convert to + 4 cells, demonstrating the extreme flexibility of tissue repair mechanisms in the intestine. Due to the similarities in marker expression and maintenance environment between ISCs and hPSC-LSCs identified in our study, it is tempting to speculate the existence of similar populations in the limbus.

**Fig. 6:**
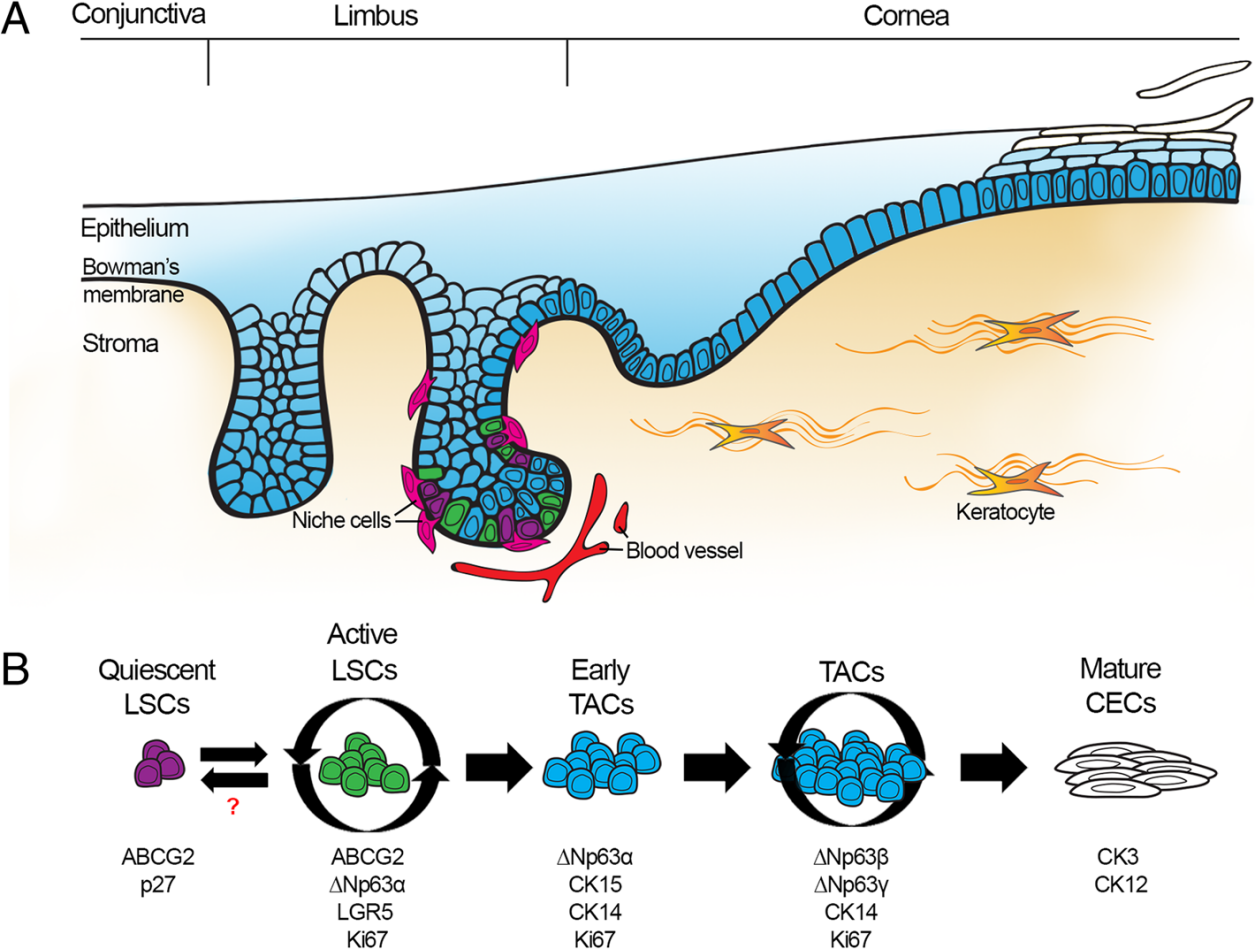
Proposed differentiation hierarchy of the LSC populations in the human cornea**. a** Schematic illustration of the human limbus, proposing that populations of both quiescent (purple) and actively cycling (green) LSCs coexist in the limbal crypts, from where their TAC (blue) progeny migrate towards the central cornea, going through several cell divisions before terminal differentiation into mature (white) CECs. **b** Representation of the interplay between separate limbal cell populations, identified by distinct marker expression profiles. LSC limbal stem cell, TAC transiently amplifying cell, CEC corneal epithelial cell

To summarize, during the differentiation of hPSC-LSCs, we were able to identify three distinct populations of (1) ABCG2-positive/∆Np63α-negative quiescent cells, (2) cells with stem cell characteristics expressing ABCG2 and LGR5 with “dim” ∆Np63α expression and a moderate proliferation rate upon ENRC maintenance, and (3) a later emerging population of highly proliferative ∆Np63α/CK14/CK15-expressing cells, which could represent an early unspecified TAC phenotype (Fig. 6). A deeper understanding of the functional roles of these stem cell populations and their in-depth molecular characterization may be utilized to unravel the functions of different LSC phenotypes and the mechanisms of corneal regeneration. Overall, these in vitro findings suggest the heterogenic and/or plastic nature of limbal progenitors and the potential concomitant operation of both quiescent and actively proliferating stem cell compartments in the cornea. From a translational point of view, it is important to consider the role of cellular quiescence in therapeutic efficacy, as it has been speculated whether stem cell therapies containing quiescent cells might prove beneficial over approaches which mainly include transplantation of proliferating LSCs and their progeny [38, 46]. Thus, for ocular surface applications such as treatment for LSCD, a graft that also incorporates quiescent cells might better endure transplantation-related cellular stress, leading to improved long-term efficacy. This hypothesis was highly supported by our data from the ex vivo transplantations into the porcine cornea model that demonstrated superior regenerative potential of the ABCG2-positive hPSC-LSCs over the ∆Np63α-positive population. Importantly, because ABCG2 identified both the quiescent and proliferating hPSC-LSC populations, it could be envisioned as a selection marker for new hPSC-based therapeutic strategies to treat corneal blindness caused by LSCD.

## Conclusions

In summary, this article describes an extensive characterization of the corneal differentiation of human pluripotent stem cells as well as a novel method to maintain the stemness of the functional differentiated limbal stem cells in culture. The study indicates new relationships in the limbal stem cell hierarchy and identity, providing tools for further development of safe and more efficient therapies for LSCD.

## Additional files

Additional file 1: Full detailed descriptions for the hPSC differentiation towards LSCs and standard hPSC-LSC culture, establishment and maintenance of the ABCG2-positive hPSC-LSC culture, immunofluorescence characterization protocol, flow cytometry analysis and fluorescence-activated cell sorting protocol, quantitative RT-PCR protocol, and cell surface antigen screening with the LEGENDScreen™ Kit. (DOCX 29 kb)

Additional file 2: **Table S1.** Primary and secondary antibodies used in this study. (DOCX 22 kb)

Additional file 3: **Figure S1.** Description of three distinct stem cell population phenotypes emerging during the hPSC-LSC differentiation, separated by their expression patterns for ABCG2 and ∆Np63α. Representative cells from different populations are marked with symbols to the IF images of d10 and d24 cytospin samples. Scale bars, 100 μm. Cell nuclei counterstained with DAPI (blue). Data are presented with the representative hESC line Regea08/017. (DOCX 742 kb)

Additional file 4: **Figure S2.** (A) Representative flow cytometry graphs of the negative controls, isotype controls, and ABCG2-stained hPSC-LSC samples in different time points. (B) Morphology and ABCG2/p63α expression of day 11 sorted ABCG2-positive hPSC-LSCs after continued culture (17 days) in CnT-30 medium and on LN-521/Col IV (B). Scale bar, 100 μm. Cell nuclei counterstained with DAPI (blue). BF: brightfield, FACS: fluorescence-activated cell sorting. Data are presented with the representative hESC line Regea08/017. (DOCX 1668 kb)

Additional file 5: **Figure S3.** Characterization of putative LSC marker expression during hPSC-LSC differentiation for hiPSC line UTA.04607.WT. (A) Representative morphology and protein expression of the cultures at selected time points. Scale bars, 100 μm for all images in the same column. Cell nuclei counterstained with DAPI (blue). (B) Marker expression differences in the d10 and d24 populations. Five images per sample and a minimum of 600 cells per time point were analyzed for each marker from cytospin samples. (C) p63α and ABCG2 expression in d10 and d24 hPSC-LSCs. Five images per sample and a minimum of 3 000 cells per time point were analyzed from cytospin samples. (D) The level of ABCG2 protein expression in UD-hPSCs and in d10 and d24–26 hPSC-LSCs, analyzed with flow cytometry. (G) The ABCG2 mRNA expression levels in UD-hPSCs and in d10 and d24 hPSC-LSCs analyzed with qRT-PCR. All quantitative data are presented as the mean + SD and *n* marks the individual cell differentiation batches serving as biological replicates. Statistical analysis in (D) was carried out using the Mann-Whitney *U* test. *P ≤ 0.05. (DOCX 4413 kb)

Additional file 6: **Table S2.** Distribution of ABCG2-positive and ABCG2-negative cells in Regea08/017 d10 hPSC-LSC subpopulations marked by selected cell surface markers. (DOCX 19 kb)

Additional file 7: **Figure S4.** (A) Additional characterization of OCT3/4, PAX6, CK14, and CK15 expression at d24 in the novel CnT-07+ENRC maintenance condition. (B) Characterization of Ki67/ABCG2 protein expression at d11, as well as after continued culture in CnT-07+ENRC or CnT-30 at d21. In both panels, cell nuclei counterstained with DAPI (blue) and scale bars, 100 μm. Data are presented for the hESC lines Regea11/013 (A) and Regea08/017 (B). (DOCX 5938 kb)

## Data Availability

All the data used to support the findings of this study are included within the article and its supplementary files.

## Abbreviations

ABCB5: ATP-binding cassette sub-family B member 5
ABCG2: ATP-binding cassette super-family G member 2
bFGF: Basic fibroblast growth factor
BMI-1: Polycomb complex protein BMI-1
BMP: Bone morphogenetic protein
CD: Cluster of differentiation
CDK: Cyclin-dependent kinase
CE: Corneal epithelium
CK: Cytokeratin
CLET: Cultured limbal epithelial transplantation
Col IV: Collagen type IV
d: Day
EB: Embryoid body
EGF: Epidermal growth factor
ENRC: Growth factor combination of EGF, Noggin, R-spondin-1, CHIR99021
FBS: Fetal bovine serum
HE: Hematoxylin and eosin stain
hiPSC: Human induced pluripotent stem cell
hPSC: Human pluripotent stem cell
hPSC-LSC: Human pluripotent stem cell-derived limbal stem cell
IF: Immunofluorescence
ISC: Intestinal stem cell
LGR5: Leucine-rich repeat-containing G-protein coupled receptor 5
LN-521: Recombinant laminin-521
LSC: Limbal stem cell
LSCD: Limbal stem cell deficiency
NaOH: Sodium hydroxide
p: Passage
PD: Population doubling
PDT: Population doubling time
qRT-PCR: Quantitative reverse transcription polymerase chain reaction
SD: Standard deviation
TAC: Transient amplifying cell
UD: Undifferentiated
