# Supplementary material for: Modulation of Wnt/BMP pathways during corneal differentiation of hPSC maintains ABCG2-positive LSC population that demonstrates increased regenerative potential
Source: Stem Cell Res Ther. 2019 Aug 5;10:236. doi: 10.1186/s13287-019-1354-2 (PMC6683518; doi:10.1186/s13287-019-1354-2)
Supplement: Supplementary file 1 — Full detailed descriptions for the hPSC differentiation towards LSCs and standard hPSC-LSC culture, establishment and maintenance of the ABCG2-positive hPSC-LSC culture, immunofluorescence characterization protocol, flow cytometry analysis and fluorescence-activated cell sorting protocol, quantitative RT-PCR protocol, and cell surface antigen screening with the LEGENDScreen™ Kit. (DOCX 29 kb) [file 13287_2019_1354_MOESM1_ESM.docx]

**SUPPLEMENTAL MATERIALS AND METHODS**

**hPSC differentiation towards LSCs and standard hPSC-LSC culture**

The differentiation method used in this study has been previously described in full in Hongisto et al. (2017). In brief, undifferentiated (UD-)hPSCs were initially transferred from standard culture on human foreskin fibroblast (hFF) feeder cells to feeder cell-free conditions and thereafter cultured on well plates coated with 0.55 μg/cm2 recombinant laminin-521 (LN-521; Biolamina, Sweden) in Essential 8™ Flex Medium supplemented with 50 U/ml penicillin-streptomycin (both from Thermo Fisher Scientific). Single-cell passaging was carried out twice a week using xeno-free TrypLE™ Select Enzyme (TrypLE; Gibco, Thermo Fisher Scientific) and initial plating density of 40 000-50 000 cells/cm^2^. Only high-quality feeder-free hPSCs expanded with this method no further than 15 passages were used in the experiments.

Similarly, the differentiation of feeder-free hPSCs towards the corneal lineage was carried out following the method in Hongisto et al. (2017). Details of the technique are also available as visualized protocol in Hongisto et al. (2018). To recap, colonies of subconfluent UD-hPSCs were dissociated with TrypLE and the acquired single-cell suspension was transferred onto low-attachment plate in defined XF-Ko-SR medium consisting of KnockOut™ DMEM supplemented with 15% KnockOut™ SR XenoFree CTS™, 2 mM GlutaMAX™, 0.1 mM 2-Mercaptoethanol (all from Gibco, Thermo Fisher Scientific), 1% MEM Non-Essential Amino Acids, and 50 U/ml Penicillin-Streptomycin (both from Lonza). To support the formation of EBs over the first night in the suspension culture, 5 µM blebbistatin (Sigma-Aldrich) was added to XF-Ko-SR. During the subsequent three-day surface ectodermal induction period as EBs, XF-Ko-SR medium was first supplemented with 10 µM SB-505124 and 50 ng/ml human basic fibroblast growth factor (bFGF; PeproTech Inc., Rocky Hill, NJ) for one day and with 25 ng/ml bone morphogenetic protein (BMP)-4 (PeproTech Inc.) for two days. After induction, the EBs were transferred onto wells coated with 0.5 µg/cm^2^ LN-521 and 5 µg/cm^2^ human placental collagen Type IV (Col IV, Sigma-Aldrich) in defined commercial CnT-30 corneal differentiation medium (CELLnTEC Advanced Cell Systems AG, Bern, Switzerland) supplemented with 50 U/ml Penicillin-Streptomycin for further epithelial differentiation. During standard differentiation, adherent cultures were thereafter maintained in CnT-30, with medium changes three times per week.

The hPSC-derived LSCs were used for experiments at various time points between d7 and d24 of the differentiation process. During routine cell culture, d21-24 hPSC-LSCs were cryopreserved using PSC Cryopreservation Medium (Thermo Fisher Scientific) according to the manufacturer’s instructions. In some experiments, hPSC-LSCs recovered from cryostorage were used in place of freshly differentiated d24 hPSC-LSCs. This was considered reasonable because of the practical matters related in experimental design and schedule, as we have previously shown that the corresponding phenotype is maintained in cryopreserved hPSC-LSCs after thawing (Hongisto et al., 2017). Upon thawing, hPSC-LSCs were returned to initial culture conditions on LN-521/Col IV coated well plates and allowed to recover and expand approximately 2 to 4 days in CnT-30 medium before using them for experiments.

**Establishment and maintenance of the ABCG2-positive hPSC-LSC culture**

Standard culturing under CnT-30 conditions resulted in loss of the colony morphology and ABCG2 expression and promoted fast differentiation of hPSC-LSCs towards ∆Np63α-positive epithelial monolayers, as described for the hPSC-LSC differentiation process. In addition, the proliferative capacity of hPSC-LSCs rapidly diminished upon passaging in CnT-30, and proliferation generally ceased after the third passage, as we have repeatedly witnessed during our CnT-30-based cell culture routine using various cell lines. Hence, alternative culture conditions to delay or prevent loss of the ABCG2 expression were tested.

Replacing the corneal differentiation medium CnT-30 with the commercial epithelial maintenance medium CnT-07 from the same company, CellNTec, only slightly extended the timeframe during which ABCG2-positive hPSC-LSC colonies were observed (data not shown), thus additional supplementation options to regulate the differentiation process was explored. CnT-07 was further supplemented with **ENRC** (50 ng/ml mouse recombinant epidermal growth factor (**E**GF, Invitrogen), 100 ng/ml mouse recombinant **N**oggin, 1 µg/ml human recombinant **R**-spondin (both from PeproTech) and 3 µM **C**HIR -99021 (Stemgent)) and the new medium was changed to the cells approximately at d10 of differentiation.

ENRC medium was introduced directly to the adherent cultures; alternatively, the hPSC-LSCs were concomitantly dissociated to a single-cell suspension using TrypLE and passaged onto fresh LN-521/Col IV-coated wells at a density of 1 000 cells/cm^2^ in the new medium. Human PSC-LSCs were thereafter cultured following the standard feeding regimen, changing the ENRC medium three times per week. After the emergence of ABCG2-positive colonies, expansion of hPSC-LSCs in ENRC was carried out by passaging subconfluent cultures onto fresh LN-521/Col IV-coated matrices at a density of 1 000 cells/cm^2^.

Two different time points were tested to determine the optimal culture phase for passaging the ABCG2-positive hPSC-LSCs. In the first approach, the first passage was carried out approximately at d10 of hPSC-LSC differentiation and the cells were subsequently introduced to the new ENRC medium, as described above. In the second approach, the first passage was carried out between d21 and d24, i.e., 10-13 days after introduction of the ENRC condition and establishment of ABCG2-positive colonies. Successful maintenance of ABCG2-positive colonies during passaging was achieved with both approaches.

**Immunofluorescence characterization protocol**

Basic IF procedure for the visualization of OCT3/4, PAX6, ABCG2, p63α, ∆Np63, CK15, CK14, CK12, LGR5 and p27 protein expression was performed essentially as described previously in Mikhailova et al. (2014). CD200 staining was carried out following the same protocol but no staining was observed in any of the attempts. Unless stated otherwise, the steps for staining were carried out at room temperature (RT). Briefly, adherent cell cultures on well plates or detached cells spun onto cover slips for cell counting analysis were fixed with 4% paraformaldehyde (PFA, Sigma Aldrich) for 15-20 min, washed three times with PBS and optionally stored at +4°C until proceeding to the staining protocol (except for the cytospin samples that were stained immediately). The cell membranes were permeabilized with 0.1% Triton X-100 (Sigma-Aldrich) for 10 min, and nonspecific binding sites were blocked with 3% bovine serum albumin (BSA, Sigma-Aldrich) for 1 hour. After blocking, the samples were incubated with appropriately diluted primary antibodies in 0.5% BSA overnight at +4°C. The next day, the samples were washed three times for 5 min with PBS and incubated with 1:800 diluted secondary antibodies in 0.5% BSA for 1 hour. Then, the samples were washed again and finally mounted with mounting medium containing DAPI (VectaShield from Vector Laboratories or ProLong Gold Antifade from Thermo Fischer Scientific). Depending on the mounting medium, the samples were immediately stored at +4°C (VectaShield) or allowed to harden and kept at RT (ProLong) until imaging, protected from the light.

**Flow cytometry analysis and fluorescence-activated cell sorting protocol**

For immunostaining with ABCG2 and CD200 FACS antibodies, the cells were first enzymatically dissociated and washed with prechilled FACS wash buffer containing 0.5 or 3.0% BSA and 2 nM EDTA (Gibco, Thermo Fisher Scientific). Optimized volumes of the appropriate antibodies were added to approximately 100 µl sample volume containing 2-10 x 10^5^ cells and incubated for 15-20 min on ice, protected from light. After incubation, the samples were washed two times, resuspended in FACS buffer and stored on ice until analysis or sorting. APC-conjugated monoclonal mouse anti-human CD338 (ABCG2) antibody, clone 5D3 (BD Pharmingen, #561451) and PE-conjugated mouse monoclonal CD200 (clone OX-104) antibodies from two manufacturers (BioLegend, #329205 and BD Pharmingen, #561762) were used for FACS analysis. An APC-conjugated mouse IgG2b κ antibody (BD Pharmingen, #555745) or unstained cells were used as isotype and/or negative controls, respectively, when applicable. Both flow cytometry analyses and cell sorting were performed using a BD FACSAria™ Fusion cell sorter (BD Biosciences, San Jose, California, USA). At least 10 000 events were recorded from the initially gated population. Further data analysis was performed with FlowJo 10 software (BD Biosciences, San Jose, California, USA).

In the sorting experiment, the cells were stained with APC-conjugated ABCG2 antibody as described above. 1 000 cells from the day 11 Regea08/017 ABCG2-positive hPSC-LSC population were sorted directly onto three LN521/Col IV-coated wells in CnT-30 medium supplemented with a 10 µM concentration of the ROCK inhibitor Y-27632 (Tocris Bioscience). After sorting, the cells were cultured following the standard feeding regimen.

**Quantitative RT-PCR protocol**

Total RNA was extracted from the cell pellets with the RNeasy Mini Kit (Qiagen, Thermo Fisher Scientific) following the instructions of the manufacturer or optionally using the TRIzol™ Reagent, followed by removal of single- and double-stranded DNA with DNase I treatment (both from Thermo Fisher Scientific). The RNA concentration of each sample was determined using a NanoDrop-1000 spectrophotometer (NanoDrop Technologies), and 400 ng total RNA from each sample was used for cDNA synthesis using the High-Capacity cDNA Reverse Transcription Kit (Applied Biosystems, Thermo Fisher Scientific). The resulting cDNA samples were analyzed for the ABCG2 mRNA with qPCR, using a sequence-specific TaqMan Gene Expression Assay (#HS01053790_m1, Applied Biosystems). GAPDH (Hs99999905_m1) was used as a housekeeping gene. All samples and controls were run as triplicate reactions with the 7300 Real-Time PCR system (Applied Biosystems). The results were analyzed using the -2^∆∆Ct^ method (Livak and Schmittgen, 2001) and are presented as the fold change in gene expression normalized to GAPDH and relative to the undifferentiated control cells (UD-hPSCs).

**Cell surface antigen screening with the LEGENDScreen™ Kit**

Additional cell surface marker screening was performed for Regea08/017 hPSC-LSCs at day 10, using the LEGENDScreen™ Lyophilized Antibody Array, Human PE Kit (BioLegend, #700007). Because of the large number of cells required for the analysis, the experiment was carried out in four parts, one plate at a time. The preparations were essentially made as instructed by the manufacturer. Briefly, lyophilized antibodies were reconstituted in their wells 1-8 days before the experiment date, after which the plates were sealed and stored at +4°C until starting the experiment. Day 10 hPSC-LSCs were dissociated using Tryple Select and DTI, counted and seeded into the multiwell plate in the kit’s own cell staining buffer, using 120 000–150 000 cells in a sample volume of 75 µl per well. Staining and fixing of the cells in the well plate were then conducted according to the manual, and at least 10,000 events from the initially gated population were recorded for each well with a FACSCanto II flow cytometer (BD Biosciences, San Jose, California, USA) and analyzed with FlowJo software.
