# Supplementary material for: Modulation of Wnt/BMP pathways during corneal differentiation of hPSC maintains ABCG2-positive LSC population that demonstrates increased regenerative potential
Source: Stem Cell Res Ther. 2019 Aug 5;10:236. doi: 10.1186/s13287-019-1354-2 (PMC6683518; doi:10.1186/s13287-019-1354-2)
Supplement: Supplementary file 2 — Table S1. Primary and secondary antibodies used in this study. (DOCX 22 kb) [file 13287_2019_1354_MOESM2_ESM.docx]

**SUPPLEMENTAL INFORMATION**

**Table S1.**

| Primary antibody | Host | Manufacturer | Catalog no | Dilution |
| --- | --- | --- | --- | --- |
| *OCT3/4* | goat | R&D | AF1759 | 1:200 |
| *PAX6* | rabbit | Sigma-Aldrich | HPA030775 | 1:200 |
| *ABCG2 (clone 5D3)* | mouse | Millipore | MAB4155 | 1:200 |
| *p63α* | rabbit | Cell Signaling Techologies | 4892 | 1:200 |
| *p40/ΔNp63* | mouse | BioCare Medical | ACI3066A | 1:100 |
| *Cytokeratin 14* | mouse | R&D | MAB3164 | 1:300 |
| *Cytokeratin 15* | mouse | Thermo/Neomarkers | MS-1068-P1 | 1:200 |
| *Cytokeratin 12* | goat | Santa Cruz Biotechnologies | SC-17099 | 1:200 |
| *LGR5* | rabbit | Abcam | ab75732 | 1:100 |
| *p27/KIP* | rabbit | Abcam | ab32034 | 1:200 |
| *CD200* | mouse | BioLegend | 329201 | 1:100 |
| Secondary antibody | **Host** | **Manufacturer** | **Order no** | **Dilution** |
| *Anti-mouse A488* | goat | Molecular Probes | A-21042 | 1:800 |
| *Anti-rabbit A488* | donkey | Molecular Probes | A-21206 | 1:800 |
| *Anti-goat A488* | donkey | Molecular Probes | A-11055 | 1:800 |
| *Anti-mouse A568* | donkey | Molecular Probes | A-10037 | 1:800 |
| *Anti-goat A568* | donkey | Molecular Probes | A-11057 | 1:800 |
| *Anti-rabbit A647* | donkey | Molecular Probes | A-31573 | 1:800 |
| *Anti-goat A647* | donkey | Abcam | ab150131 | 1:800 |
