# Supplementary figures and images for: Modulation of Wnt/BMP pathways during corneal differentiation of hPSC maintains ABCG2-positive LSC population that demonstrates increased regenerative potential
Source: Stem Cell Res Ther. 2019 Aug 5;10:236. doi: 10.1186/s13287-019-1354-2 (PMC6683518; doi:10.1186/s13287-019-1354-2)

**SUPPLEMENTAL RESULTS**

**Figure S1.**

**
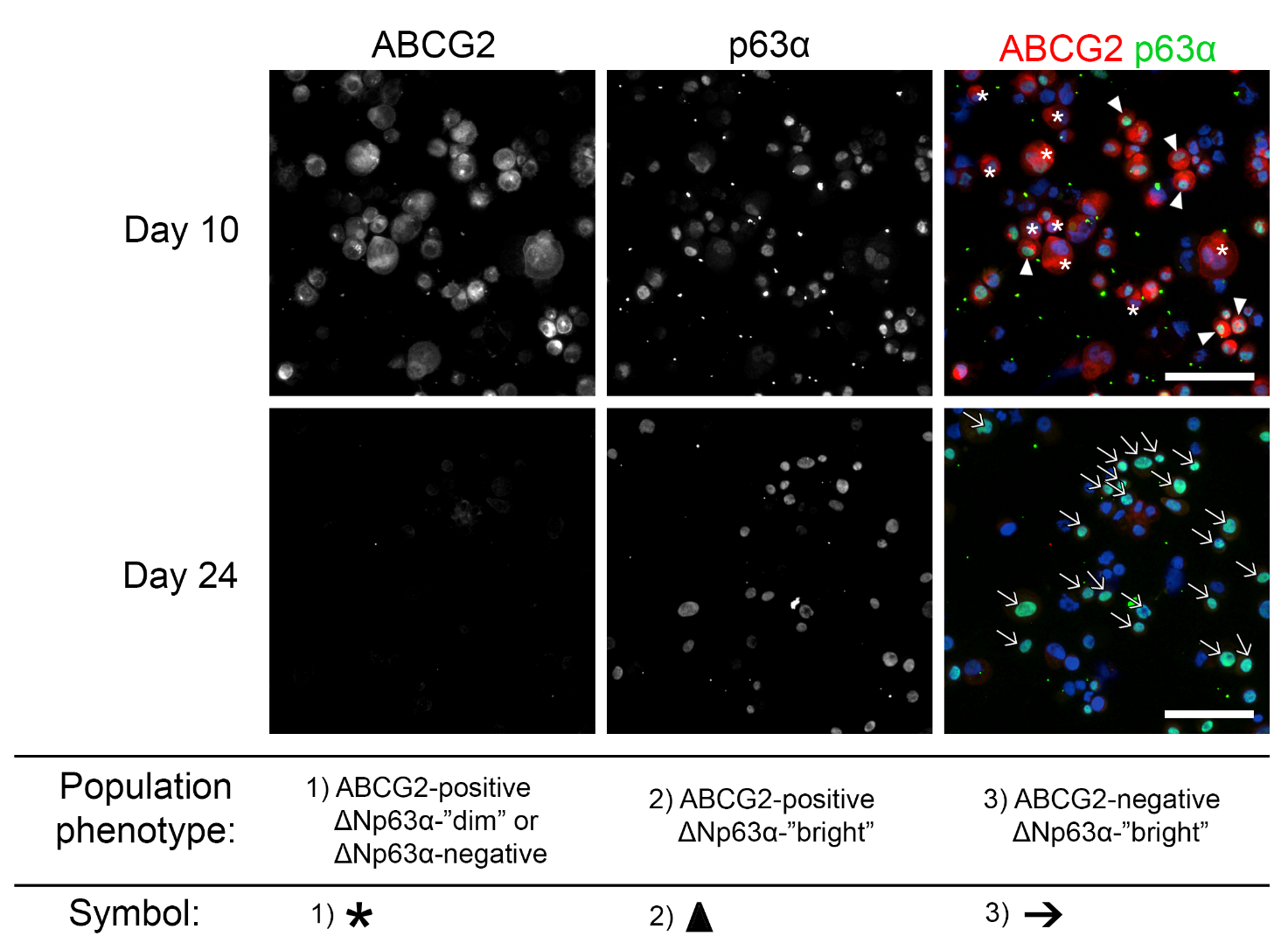
**

Supplement: Supplementary file 3 — Figure S1. Description of three distinct stem cell population phenotypes emerging during the hPSC-LSC differentiation, separated by their expression patterns for ABCG2 and ∆Np63α. Representative cells from different populations are marked with symbols to the IF images of d10 and d24 cytospin samples. Scale bars, 100 μm. Cell nuclei counterstained with DAPI (blue). Data are presented with the representative hESC line Regea08/017. (DOCX 742 kb) [file 13287_2019_1354_MOESM3_ESM.docx]

**SUPPLEMENTAL RESULTS,**

**Figure S2.**


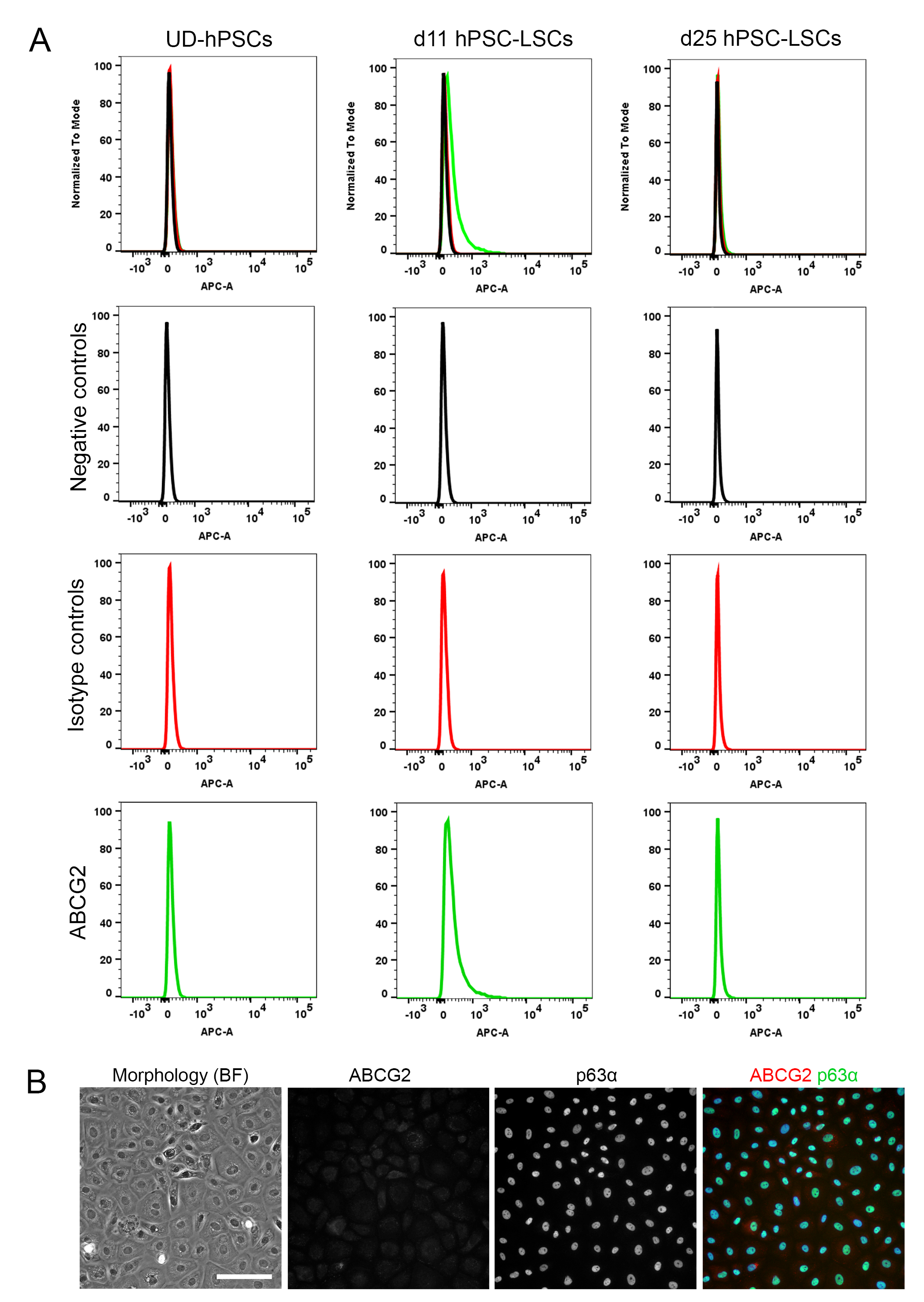

Supplement: Supplementary file 4 — Figure S2. (A) Representative flow cytometry graphs of the negative controls, isotype controls, and ABCG2-stained hPSC-LSC samples in different time points. (B) Morphology and ABCG2/p63α expression of day 11 sorted ABCG2-positive hPSC-LSCs after continued culture (17 days) in CnT-30 medium and on LN-521/Col IV (B). Scale bar, 100 μm. Cell nuclei counterstained with DAPI (blue). BF: brightfield, FACS: fluorescence-activated cell sorting. Data are presented with the representative hESC line Regea08/017. (DOCX 1668 kb) [file 13287_2019_1354_MOESM4_ESM.docx]

**SUPPLEMENTAL RESULTS**

**Figure S3.**


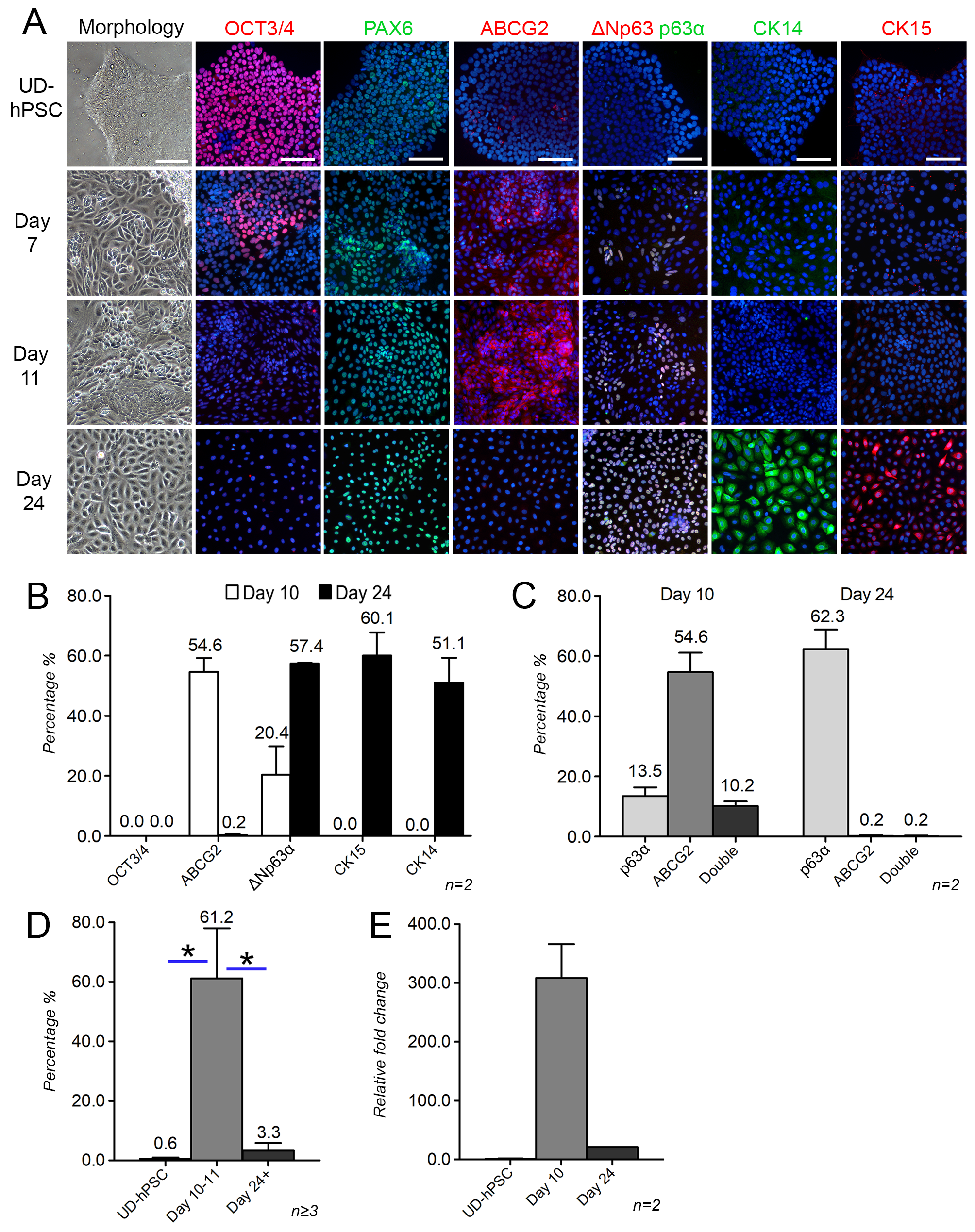

Supplement: Supplementary file 5 — Figure S3. Characterization of putative LSC marker expression during hPSC-LSC differentiation for hiPSC line UTA.04607.WT. (A) Representative morphology and protein expression of the cultures at selected time points. Scale bars, 100 μm for all images in the same column. Cell nuclei counterstained with DAPI (blue). (B) Marker expression differences in the d10 and d24 populations. Five images per sample and a minimum of 600 cells per time point were analyzed for each marker from cytospin samples. (C) p63α and ABCG2 expression in d10 and d24 hPSC-LSCs. Five images per sample and a minimum of 3 000 cells per time point were analyzed from cytospin samples. (D) The level of ABCG2 protein expression in UD-hPSCs and in d10 and d24–26 hPSC-LSCs, analyzed with flow cytometry. (G) The ABCG2 mRNA expression levels in UD-hPSCs and in d10 and d24 hPSC-LSCs analyzed with qRT-PCR. All quantitative data are presented as the mean + SD and n marks the individual cell differentiation batches serving as biological replicates. Statistical analysis in (D) was carried out using the Mann-Whitney U test. *P ≤ 0.05. (DOCX 4413 kb) [file 13287_2019_1354_MOESM5_ESM.docx]

**SUPPLEMENTAL RESULTS**

**Figure S4.**


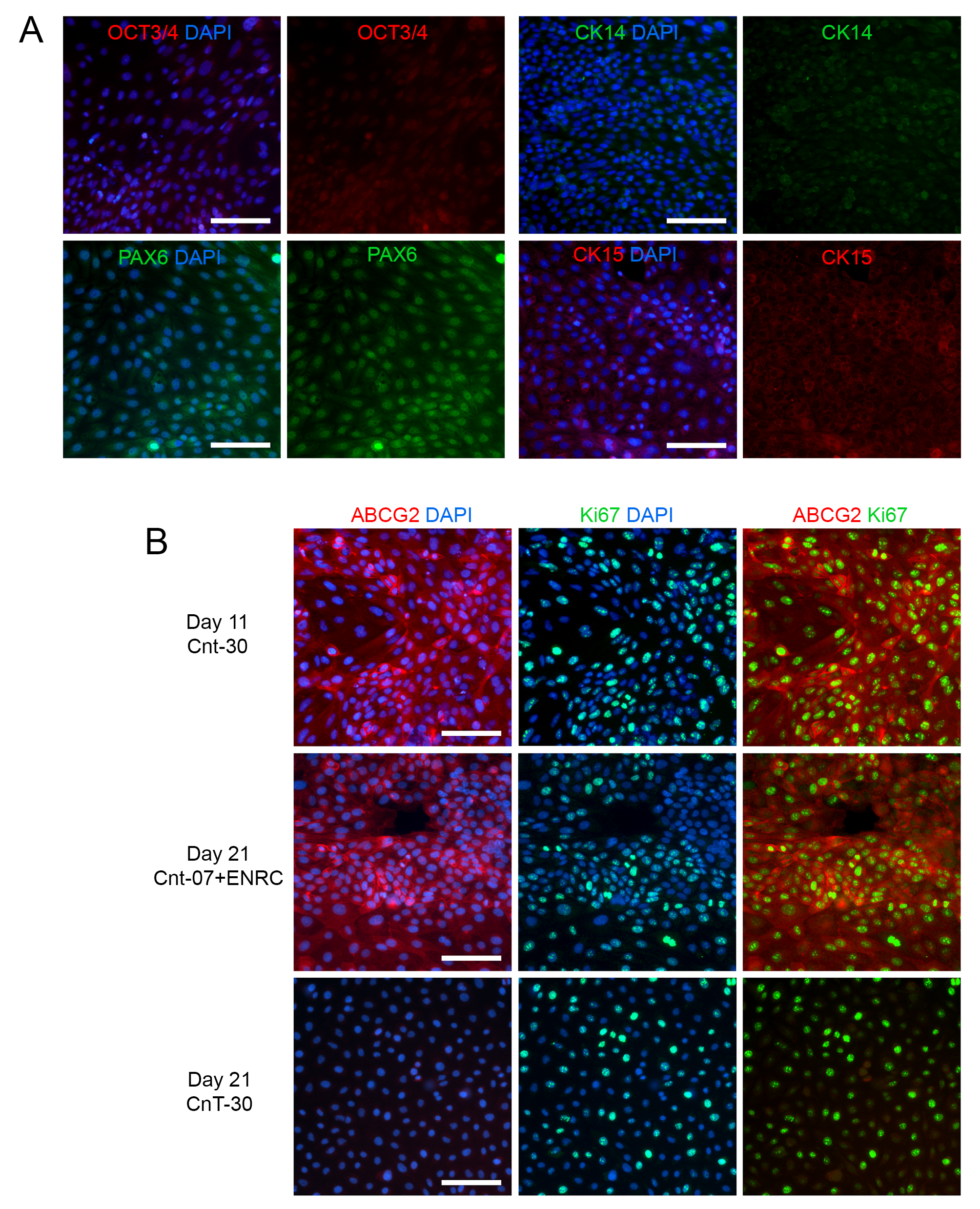

Supplement: Supplementary file 7 — Figure S4. (A) Additional characterization of OCT3/4, PAX6, CK14, and CK15 expression at d24 in the novel CnT-07+ENRC maintenance condition. (B) Characterization of Ki67/ABCG2 protein expression at d11, as well as after continued culture in CnT-07+ENRC or CnT-30 at d21. In both panels, cell nuclei counterstained with DAPI (blue) and scale bars, 100 μm. Data are presented for the hESC lines Regea11/013 (A) and Regea08/017 (B). (DOCX 5938 kb) [file 13287_2019_1354_MOESM7_ESM.docx]
