# Supplementary material for: Modulation of Wnt/BMP pathways during corneal differentiation of hPSC maintains ABCG2-positive LSC population that demonstrates increased regenerative potential
Source: Stem Cell Res Ther. 2019 Aug 5;10:236. doi: 10.1186/s13287-019-1354-2 (PMC6683518; doi:10.1186/s13287-019-1354-2)
Supplement: Supplementary file 6 — Table S2. Distribution of ABCG2-positive and ABCG2-negative cells in Regea08/017 d10 hPSC-LSC subpopulations marked by selected cell surface markers. (DOCX 19 kb) [file 13287_2019_1354_MOESM6_ESM.docx]

**SUPPLEMENTAL RESULTS**

**Table S2.**

| **Surface marker** | **ABCG2+** | **ABCG2-** |
| --- | --- | --- |
| EGFR | 59.4 % | 40.6 % |
| CD71 | 53.7 % | 46.3 % |
| Integrin β5 | 41.3 % | 58.7 % |
| Integrin α6 (CD49f) | 53.5 % | 46.5 % |
| E-cadherin (CD324) | 71.5 % | 28.5 % |
| CD40 | 69.5 % | 30.5 % |
| CD146 | 68.4 % | 31.6 % |
| CD166 | 61.2 % | 38.8 % |
| CD200 | 48.1 % | 51.9 % |
| CD109 | 58.5 % | 41.5 % |
